# Stepwise kinetic equilibrium models of quantitative polymerase chain reaction

**DOI:** 10.1186/1471-2105-13-203

**Published:** 2012-08-16

**Authors:** Gary Cobbs

**Affiliations:** 1 Department of Biology, University of Louisville, Louisville, Kentucky, 40292, USA

**Keywords:** Quantitative polymerase chain reaction, qPCR, Kinetic model

## Abstract

**Background:**

Numerous models for use in interpreting quantitative PCR (qPCR) data are present in recent literature. The most commonly used models assume the amplification in qPCR is exponential and fit an exponential model with a constant rate of increase to a select part of the curve. Kinetic theory may be used to model the annealing phase and does not assume constant efficiency of amplification. Mechanistic models describing the annealing phase with kinetic theory offer the most potential for accurate interpretation of qPCR data. Even so, they have not been thoroughly investigated and are rarely used for interpretation of qPCR data. New results for kinetic modeling of qPCR are presented.

**Results:**

Two models are presented in which the efficiency of amplification is based on equilibrium solutions for the annealing phase of the qPCR process. Model 1 assumes annealing of complementary targets strands and annealing of target and primers are both reversible reactions and reach a dynamic equilibrium. Model 2 assumes all annealing reactions are nonreversible and equilibrium is static. Both models include the effect of primer concentration during the annealing phase. Analytic formulae are given for the equilibrium values of all single and double stranded molecules at the end of the annealing step. The equilibrium values are then used in a stepwise method to describe the whole qPCR process. Rate constants of kinetic models are the same for solutions that are identical except for possibly having different initial target concentrations. Analysis of qPCR curves from such solutions are thus analyzed by simultaneous non-linear curve fitting with the same rate constant values applying to all curves and each curve having a unique value for initial target concentration. The models were fit to two data sets for which the true initial target concentrations are known. Both models give better fit to observed qPCR data than other kinetic models present in the literature. They also give better estimates of initial target concentration. Model 1 was found to be slightly more robust than model 2 giving better estimates of initial target concentration when estimation of parameters was done for qPCR curves with very different initial target concentration. Both models may be used to estimate the initial absolute concentration of target sequence when a standard curve is not available.

**Conclusions:**

It is argued that the kinetic approach to modeling and interpreting quantitative PCR data has the potential to give more precise estimates of the true initial target concentrations than other methods currently used for analysis of qPCR data. The two models presented here give a unified model of the qPCR process in that they explain the shape of the qPCR curve for a wide variety of initial target concentrations.

## Background

**Figure 1:**
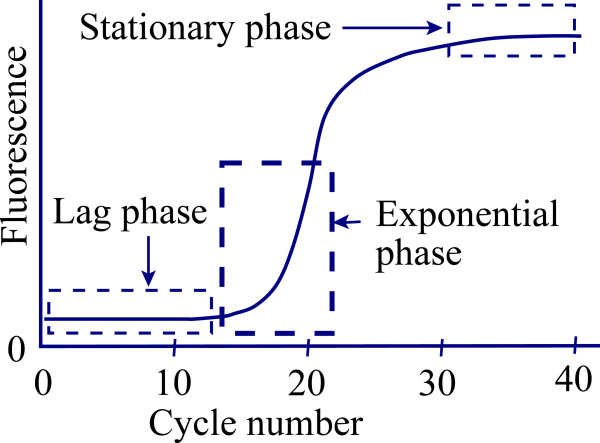
**A typical amplification curve resulting from a qPCR experiment** .

Quantitative Polymerase Chain Reaction, qPCR, has become a common tool of molecular biology to determine the absolute or relative concentrations of particular DNA sequences in samples. The method gives fluorescent values for each of a number of consecutive cycles beginning with cycle 1. Before cycle 1 the initial concentration of double-stranded DNA target sequence is T_0_ and its concentration is amplified in successive cycles to produce a high concentration of the target sequence. At each cycle, the efficiency of amplification (E) is the ratio of the amount of newly synthesized target at the end of the cycle to the amount present at the beginning, thus the amount of target at the end of the cycle is (1 + E) times the amount at the beginning. If every single-stranded target molecule re-associates with exactly one primer molecule and all these structures are extended by polymerase to completely synthesize the complementary target strand, the value of E is 1, which is its theoretical upper limit. The increase in DNA concentration is monitored by a detection system which generates fluorescence in proportion to the concentration of target DNA sequence. Several different types of detection systems are in common usage that generate fluorescence through different types of reactions. I present here two models of qPCR for use when the detection systems uses DNA binding dyes such as SYBR green or SYTO-13. During early cycles of the amplification, the concentration of target is too small to produce measurable fluorescence and is called the lag phase (Figure 1). During lag phase the concentration of target sequence increases by (1 + E) fold in every cycle, but the initial concentration of the double-stranded target sequence (T_0_) is so small that even with repeated increases of (1 + E) fold in every cycle the target concentration is not high enough to be measurable. Thus, no information on E can be obtained from the amplification curve during lag phase. When target is present in quantities sufficient to be measured, the increase in fluorescence is approximately exponential over a number of successive cycles and the reaction is said to be in the exponential phase (Figure 1). Fluorescence values during the exponential phase may be used to estimate E and most models of qPCR use only this part of the curve to estimate E and then assume the estimated value applies throughout the lag phase as well. After exponential phase, the efficiency progressively declines due to changes in the concentrations of reactants. Eventually the reaction enters stationary phase, during which E approaches zero and increases in fluorescence are minimal (Figure 1).

Models of qPCR assume total fluorescence, F, is due to two sources: Baseline fluorescence, _*b*_*F*, and Target fluorescence, _*T*_*F*, thus total fluorescence is *F* = _*b*_*F* + _*T*_*F*. Baseline fluorescence is due to all sources other than target and is assumed to be constant throughout the qPCR experiment. Target fluorescence is generated by reaction of the detection system with target sequences and is generated in proportion to target concentration. Thus, _*T*_*F* = *K*_*f*_*T* where *T* is the concentration of the double-stranded target and *K*_*f*_ is a constant that converts the target concentration to fluorescence. Total fluorescence at the end of the ***n***^th^ cycle is:

(1) $$F_{n}{=}_{b}F+K_{f}T_{n}$$

where *T*_*n*_ is target concentration at the end of the reassociation/extension step of cycle ***n***. At the beginning of the ***n***^th^ cycle, target DNA is present at concentration *T*_*n-1*_ and is replicated with amplification efficiency *E*_*n*_. Target concentration at the end of cycle ***n*** is then: $T_{n}=T_{n-1}\left(1+E_{n}\right)$

which has the solution:

(2) $$T_{n}=T_{0}\prod_{i=1}^{n}\left(1+E_{i}\right)\;\text{n}=1,2,3,\ldots$$

Since ${}_{\;T}F_{n}=K_{f}T_{n}$ then ${}_{\;T}F_{n}{=}_{T}F_{0}\prod_{i=1}^{n}\left(1+E_{i}\right)$ and

(3) $$F_{n}{=}_{b}F{+}_{T}F_{0}\prod_{i=1}^{n}\left(1+E_{i}\right)\;\text{n}=1,2,3,\ldots$$

where _*T*_*F*_*0*_ is the amount of fluorescence that could be produced by the target sequence before cycle 1. Values of *E*_*n*_ are defined as $E_{n}=\frac{T_{n}}{T_{n-1}}-1$ for *n* = 1, 2, . . . and if equation 1 holds then *E*_*n*_ may be estimated from the observed fluorescence values as:

(4) $$E_{n}=\frac{F_{n\;-\;b}F}{F_{n-1\;-\;b}F}-1\;\text{for}\;n=1,2,3,..$$

Equation 4 is useful when *F*_*n*_ can be reliably distinguished from _*b*_*F,* which is possible only during exponential phase and later. Models of qPCR describe how *E*_*n*_ changes with ***n*** and use equation 2 to determine the target concentration or equation 3 to determine target fluorescence at each step of the process. Some models of qPCR model _*T*_*F*_*n*_ instead of *T*_*n*_ and thus use the estimate of _*T*_*F*_*0*_ instead of *T*_*0*_[1]. The estimated _*T*_*F*_*0*_ values can be converted to *T*_*0*_ by dividing by *K*_*f*_ when a value for *K*_*f*_ is available. The primary goal in analysis of qPCR data is to estimate *T*_*0*_ or _*T*_*F*_*0*_ from the qPCR curve as accurately as possible. Frequently, two or more qPCR curves are analyzed with a goal of determining the value of the ratio of two *T*_*0*_ values when *K*_*f*_ is not known. The ratio of two *T*_*0*_ values is estimated by the ratio of two _*T*_*F*_*0*_ values provided *K*_*f*_ values are the same in the two qPCR experiments. This principle is often used in qPCR analyses to compare two or more samples by estimating the ratio of two *T*_*0*_ values by the ratio of two estimated _*T*_*F*_*0*_ values [2-4].

Numerous approaches to modeling the qPCR process are present in recent literature and involve three different general approaches. First the C_t_ and Linear Regression approaches assume constant efficiency and estimate it by linear regression of ln(*F*_*n*__*b*_*F*) on cycle number using only values from the exponential phase of the curve (sometimes called the ‘Window-of-Linearity’) [3,5-9] . Second, the sigmoidal function approach has used to fit a variety of sigmoidal functions to approximate the qPCR curve (Figure 1) and estimate _*T*_*F*_*0*_ as the intercept of a sigmoidal function [1,10,11]. The sigmoidal functions used are described in the literature and none are based on a mechanistic model of the reactions in qPCR. Thirdly, kinetic models are a mechanistic approach to modeling the process and several such models have been proposed [8,12-14]. The work I present here extends the kinetic approach to modeling qPCR by including primer concentration in the model and providing analytical equilibrium solutions for the re-association phase. I also introduce the use of simultaneous analysis of qPCR curves which have common values for rate constants in the kinetic models.

### Current models

The models presented here may be regarded as an extension of the models presented by Smith *et*. *al*. (2007) [13] and Boggy and Woolf (2010) [14]. Though the models presented here are similar to those of Smith *et. al.* (2007) [13] for hydrolysis probe detection systems, they differ in that they are designed for intercalating dye detection systems such as SYBR green. They also use analytical solutions for equilibrium concentrations in the annealing step. One of the models is the same as that of Boggy and Woolf (2010) [14], however, a more general solution for equilibrium values for the annealing step is presented here that includes primer concentration and gives improved fit to data. Two stepwise kinetic equilibrium models are presented in which efficiency of amplification at each cycle depends on the concentrations of target and primers at that cycle. Events in a single cycle are modeled and then used in iterations to model the entire process. In each cycle, the re-association and the primer extension steps are modeled separately and consecutively. Double-stranded target DNA is denoted ***A***_***1***_***A***_***2***_ where ***A***_***1***_ and ***A***_***2***_ are complementary single strands. Primer that hybridizes with ***A***_***1***_ is denoted ***a***_***1***_ and primer for ***A***_***2***_ is denoted ***a***_***2***_. Primer-template hybrid molecules are denoted ***A***_***1***_***-a***_***1***_ and ***A***_***2***_ -***a***_***2***_, respectively.

### Re-association step

At the end of the dissociation step of cycle *n*, the concentration of all double-stranded structures is zero and concentrations of single-stranded molecules ***A***_***1***_, ***A***_***2***_, ***a***_***1***_, ***a***_***2***_ are *S1*_*n,0*_, *S2*_*n,0*_, *P1*_*n,0*_ and *P2*_*n,0*_, respectively, with units of concentration being moles/liter. At the end of the re-association step, the concentrations of ***A***_***1***_, ***A***_***2***_, ***a***_***1***_, ***a***_***2***_, ***A***_***1***_***-a***_***1***_***A***_***2***_-***a***_***2***_ and ***A***_***1***_-***A***_***2***_ are assumed to be at their equilibrium values *S1*_*n,e*_, *S2*_*n,e*_, *P1*_*n,e*_, *P2*_*n,e*_, *Q1*_*n,e*_, *Q2*_*n,e*_, and *D*_*n,e*_, respectively. Two different kinetic models are each used to obtain these equilibrium concentrations as a function of the initial concentrations *S1*_*n,0*_, *S2*_*n,0*_, *P1*_*n,0*_ and *P2*_*n,0*_. The initial target is entirely double-stranded at the beginning of cycle 1, so the two complementary target strands, ***A***_***1***_ and ***A***_***2***_, are initially present in equal concentrations. Primers, ***a***_***1***_ and ***a***_***2***_ are assumed to be present in equal concentrations at the beginning of cycle 1, equally effective in forming double-stranded structure with the target sequences, and equally effective in initiating synthesis with the polymerase. Under these assumptions, *S1*_*n,e*_ = *S2*_*n,e*_ = *S*_*n,e*_, *P1*_*n,e*_ = *P2*_*n,e*_ = *P*_*n,e*_, and *Q1*_*n,e*_ = *Q2*_*n,e*_ = *Q*_*n,e*_ for all ***n*** (Table 1).

**Table 1:** Summary of initial and equilibrium values for state variables of Model 1 and Model 2

**At end of:**	***A***_***1***_	***A***_***2***_	***a***_***1***_	***a***_***2***_	***A***_***1***_ -***a***_***1***_	***A***_***2***_ -***a***_***2***_	***A***_***1***_ -***A***_***2***_
dissociation step	*S*_*n,0*_	*S*_*n,0*_	*P*_*n,0*_	*P*_*n,0*_	0	0	0
re-association step	*S*_*n,e*_	*S*_*n,e*_	*P*_*n,e*_	*P*_*n,e*_	*Q*_*n,e*_	*Q*_*n,e*_	*D*_*n,e*_

### Model 1 reversible re-association

**Figure 2:**
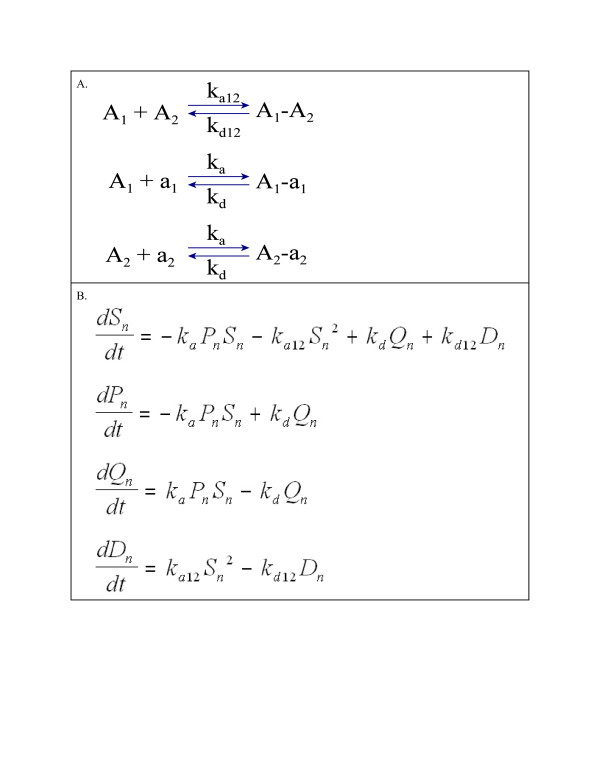
**Panel A: Dissociation/re-association reactions and rate constants for Model 1. Panel B**: Differential equations for model 1.

Reversible dissociation/re-association reactions and rate constants are shown in Figure 2A. Formation of ***A***_***1***_***-a***_***1***_***A***_***2***_-***a***_***2***_ and ***A***_***1***_-***A***_***2***_ from ***A***_***1***_, ***A***_***2***_, ***a***_***1***_, ***a***_***2***_ during the re-association step is assumed to follow the law of mass action and described by the rate equations given in Figure 2B. At equilibrium, all net rates are zero and equilibrium values of the state variables *S*_*n,e*_, *P*_*n,e*_, *Q*_*n,e*_ and *D*_*n,e*_ and are found as the simultaneous solution of equations in Figure 2B when each equation is set equal to zero. Through a series of substitutions and rearrangements of these equations (see Additional file 1), a cubic polynomial in *S*_*n,e*_ is obtained with coefficients containing only rate constants and initial concentrations S_n,0_ and P_n,0_ and is given as equation A1.6 in Additional file 1. The relevant root of the cubic equation is found using the cubic formula given in equation A1.7 of Additional file 1 and equilibrium values for the remaining state variables may be found by substituting the value of *S*_*n,e*_ into equations also given in Additional file 1. Thus *S*_*n,e*_, *P*_*n,e*_, *Q*_*n,e*_ and *D*_*n,e*_ are expressed as a known function of the rate constants k_a12_, k_d12_, k_a_, and k_d_ and the initial concentrations *S*_*n,0*_ and *P*_*n,0*_.

### Model 2 non-reversible re-association

**Figure 3:**
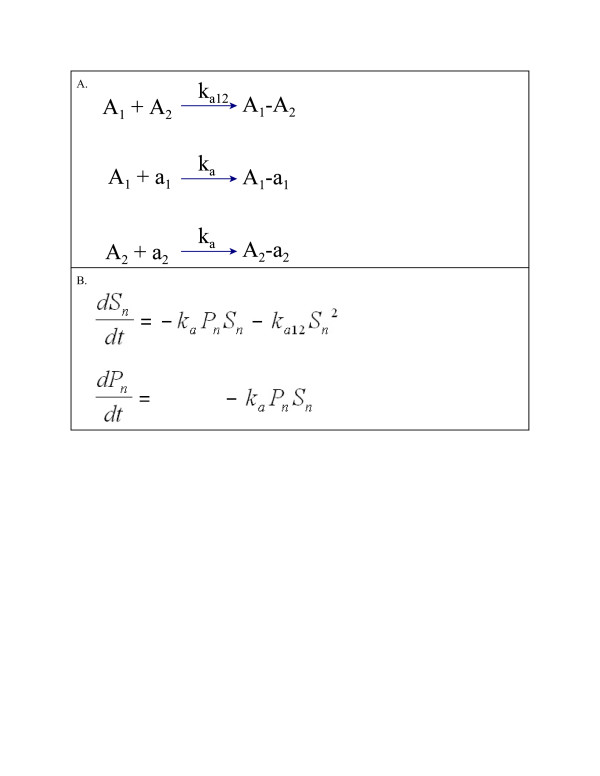
**Panel A: Dissociation/re-association reactions and rate constants for Model 2. Panel B**: Differential equations for model 2.

Non-reversible re-association reactions and rate constants are shown in Figure 3A. Reactions are again assumed to follow the law of mass action and rate equations are given in Figure 3B. At equilibrium, all net rates are zero; however, equilibrium values of the state variables *S*_*n,e*_, *P*_*n,e*_ cannot be found as the simultaneous solution of equations in Figure 3B when set equal to zero. Since *k*_*a*_ > 0, *k*_*a12*_ > 0, *P*_*n*_ ≥0 and *S*_*n*_ ≥0 then *S*_*n,e*_ = 0 is the only possible equilibrium solution and with *S*_*n,e*_ = 0, any value of *P*_*n*_ will satisfy the equilibrium equations. This result is also expected on purely scientific grounds as annealing without dissociation will always eventually reduce the single strand template concentration to a concentration of zero. The equilibrium value, *P*_*n,e*_, is found by expressing the rate of change in *P*_*n*_ as a function of *S*_*n*_ and then integrating over *S*_*n*_ as it goes from *S*_*n,0*_ to zero. The solutions for *P*_*n,e*_*, Q*_*n,e*_ and *D*_*n,e*_ are given as equations A2.4, A2.5 and A2.6, respectively, in Additional file 2 which are all known functions of *k*_*a*_, *k*_*a12*_*, S*_*n,0*_ and *P*_*n,0*_ (see Additional file 2).

### Extension step

The proportion of target/primer duplexes ***A***_***1***_***-a***_***1***_ and ***A***_***2***_-***a***_***2***_ that produce full length products in the primer extension step depends on the concentration of the polymerase and the concentration of dNTP’s. If both are in sufficiently high concentration the proportions will be near 1, however, if either or both are sufficiently low the probabilities will be less than 1. Here it will be assumed there is always sufficient polymerase and dNTP concentration to ensure complete extension of all primers in target/primer duplexes. With this assumption, at the beginning of the next cycle the concentrations of ***A***_***1***_ and ***A***_***2***_ are each given by equation 5.1 and the concentrations of ***a***_***1***_ and ***a***_***2***_ are each given by equation 5.2.

(5.1) $$S_{n+1,0}=S_{n,0}+Q_{n,e}\;n=1,2,..$$

(5.2) $$P_{n+1,0}=P_{n,0}-Q_{n,e}\;n=1,2,..$$

### The whole process

The whole qPCR process is modeled by iteration of equations 5.1 and 5.2 with *Q*_*n,e*_ determined by equation A1.9 for model 1 or equation A2.5 for model 2 (see Additional files 1 and 2). Before cycle 1, the concentrations of single-stranded target and primer are *S*_*0,0*_ and *P*_*0,0*_, respectively. The Efficiency of amplification for single stranded template at cycle *n* is (*S*_*n+1,0*_ /*S*_*n,0*_)-1 which may be obtained from equation 5.1 as

(6) $$E_{n}=\left(\frac{Q_{n,e}}{S_{n,0}}\right)\;\text{n}=1,2,3,\ldots$$

and fluorescence at cycle n is found using equation 1 as

(7) $$F_{n}{=}_{b}F+K_{f}\left(2Q_{n,e}+D_{n,e}\right)\;\text{n}=1,2,3,\ldots$$

Since equation 7 is defined only for n = 1,2,3… it cannot be used directly to obtain a value of _*T*_*F*_*0*_, however, fitting equation 7 to an actual qPCR curve provides an estimate of *S*_*0,0*_ which is the initial concentration of each of ***A***_***1***_ and ***A***_***2***_. If the initial target sample is all double-stranded then *S*_*0,0*_ is also the concentration of the double-stranded molecules, so T_0_ = S_0,0_. Defining _*T*_*F*_*0*_ as K_f_*S*_*0,0*_ provides a measure of total initial target concentration in fluorescence units. This is the amount of fluorescence that would be due to the target if it is all double-stranded. If _*T*_*F*_*0*_ could actually be measured it would be *K*_*f*_*D*_*o,e*_ where *D*_*o,e*_ is the concentration of double-stranded target DNA before cycle 1. Most likely nearly all of the target is double-stranded before cycle 1 but, since the total amount of target is what is desired using K_f_*S*_*0,0*_ as the estimate of _*T*_*F*_*0*_ is preferred.

### Relating Model 2 to Boggy & Woolf (2010)

Model 2 annealing kinetics have been analyzed by Boggy & Woolf [14] who give an approximate equilibrium solution for the re-association step and use it to obtain a recurrence formula for double stranded target concentration over successive cycles. Their approximation is obtained by removing primer concentration from the model to obtain a simpler set of differential equations which they solve to get an approximate solution. I show in Additional file 3 that my solution for model 2 becomes equivalent to theirs if $\frac{k_{a}}{k_{a12}-k_{a}}$ is small and their parameter k is set to $\left(\frac{k_{a}}{k_{a12}-k_{a}}\right)P_{n,e}$. This result indicates the rate constant, k, for the approximate solution found by Boggy & Woolf [14] depends on primer concentration and ignoring primer concentration in the analysis will cause at least some error in parameter estimates. Boggy and Woolf (2010) [14] note that their parameter k varied from one analysis to another, a result they did not expect, and this is likely due to variations in the amount of primer available. This finding suggest the analysis for model 2 presented here is a more accurate solution for model 2 kinetics than that of Boggy and Woolf (2010) [14] process since it includes variation in primer concentration.

### Restricting model fit to only part of the qPCR curve

Since models 1 & 2 both include the effect of declining primer concentration during PCR they predict a different shape of the qPCR curve between lag and stationary phase for solutions that differ in primer concentration. Consider two solutions, one with low *S*_*0,0*_ and one with high *S*_*0,0*_ and with identical primer concentrations. Allow the one with low *S*_*0,0*_ to undergo enough cycles so its current target concentration is equal to the initial target concentration for the solution with higher *S*_*0,0*_ . If primer concentration is not limiting in any way then the two solutions would have identical qPCR curves from that point on. If primer concentration is limiting in some way then the qPCR curve for the solution with lower *S*_*0,0*_ will at that point rise more slowly than that for the solution with higher S_0,0_ due to lowered primer concentration. When fitting models 1 and 2 to data it is thus desirable to include as much of the exponential phase as possible to allow changing primer concentrations to affect the analysis. Including most of the exponential phase is desired, however, in late exponential phase effects not included in the model also begin to occur and may cause the model to have poor fit. Effects in late exponential phase not modeled include reduced amount of dNTPs, reduced amount of polymerase due to decay and possibly partially replicated templates. In order to restrict analyses to only part of the exponential phase, models were fit to qPCR curves for cycles in the lag and exponential phase for which (F_n_ - _b_F)/(F_max_ - _b_F) ≤ L, where _b_F, and F_max_ are baseline fluorescence and maximum fluorescence achieved in stationary phase. The value, L, is the proportion of increase of F_n_ above _b_F and is a cut off value used to restrict the analysis to the first parts of the qPCR curve up to a point in the exponential phase where the model is thought to be valid. When choosing L, a compromise between these opposing effects is necessary and is done here by choosing the highest value for L that allows good fit to qPCR curves. To accomplish this, the models were fit to qPCR curves using a range of L values from 0.1 to 0.98 and the goodness of fit and estimated initial concentration determined for each L value. Both model 1 and 2 generally gave poor fits for L ≥ 0.95. The value of 0.8 was chosen for presentation here because the MSresidual values for most qPCR curves increased for values of L higher than 0.8 and also because the accuracy of estimates of initial target concentration was better for L values near 0.8. The question of how much of the qPCR curve to use in analysis is a problem inherent to most methods used for interpretation of qPCR curves. Boggy and Woolf (2010) [14] choose the point of maximal increase in fluorescence as their cut-off point for all curves which corresponds roughly to a value of 0.5 for L. Their rational for using only about half the exponential phase is that the effects of declining primer concentration are not likely to affect the shape of this part of the curve. The C_t_ method uses a different cut-off for every curve which is invoked when the ‘window-of-linearity’ is chosen.

### Methods for incorporating experimental design into the data analysis

Kinetic models of qPCR allow more sophisticated and powerful analyses than are possible with other models. If a group of solutions have exactly the same chemical components except for possibly differing in initial target concentration, then all the kinetic parameters should be the same for all the solutions. The rate constants may then be estimated by fitting several or many qPCR curves simultaneously to get better estimates of rate constants and initial target concentrations. In such fits the kinetic parameters are common to all the different qPCR curves in the analysis but each curve may have a unique value for initial target concentration. Also kinetic models such those presented here allow an analyst to adjust the model to accommodate different experimental designs. I present four different estimation methods (A, B, C, and D) which are used here to fit models to qPCR data which have different experimental designs.

### Standard curve method (Method A)

This method may be used only when the true initial target concentrations are known for each curve in the analysis (a standard curve). All initial conditions of the qPCR, other than initial target concentration are assumed to be the same for all curves. All curves are analyzed simultaneously in a non-linear fit in which the estimated rate constants apply to all curves and the initial target concentration is held constant at its known value for each curve. The fitting process minimizes the cost function over all cycles in all curves. The advantage of this method is that it pools the information of all the curves to give a single best estimate for each model parameter. The predicted fluorescence values provided by the analysis may be compared to the observed fluorescence values to assess how well each qPCR curve is fit by the model. The value of this method is that it may be used for estimation of initial target concentration for samples with unknown initial target concentration provided all other conditions of the PCR are the same as those used in the standard curve. This is done by doing a non-linear fit to the qPCR curve of the unknown sample in which only the initial target concentration, *S*_*0,0*_ , is estimated while using the estimated parameters from the standard curve analysis as constants. To assess how well the models presented here estimate *S*_*0,0*_ , each qPCR curve in the known samples was treated as an unknown sample and the estimated *S*_*0,0*_ value compared to the known value for *S*_*0,0*_.

### Dilution curve method (Method B)

This method may be used when a standard curve is not available but a dilution series of the unknown sample is available. Here the absolute target concentration in the undiluted sample is unknown and denoted *S*_*0,0,max*_. The absolute target concentration of the i^th^ diluted sample is d_i_*S*_*0,0,max*_ where d_i_ is the dilution ratio for the i^th^ sample. The qPCR curves from the diluted samples are analyzed simultaneously using non-linear curve fitting. Rate constants apply to all curves in the analysis and a single concentration parameter *S*_*0,0,max*_ is estimated with the initial concentration for each sample set at d_i_*S*_*0,0,max*_. In general usage of this method the estimated *S*_*0,0,max*_ would be the end result. To assess the accuracy of this method in this study the known absolute target concentrations for the data sets used here are ignored but their ratios are used to obtain values for d_i_. Simultaneous fitting of all the curves was done as described above to estimate *S*_*0,0,max*_ and all the rate constants. Next the estimated rate constants are treated as constants in a second non-linear fit of each qPCR curve separately in which an *S*_*0,0*_ value is estimated for qPCR curve. These estimated *S*_*0,0*_ values are compared to the known values to assess the accuracy of method B.

### Simultaneous curves method (Method C)

This method may be used when two or more samples are to be analyzed that may have different initial concentrations and a standard curve is not available nor are any dilutions of either sample available. The samples are assumed to be subject to qPCR with identical conditions except for the fact they may have different initial target concentrations. In this method all samples are analyzed simultaneously with estimated rate constants applying to all samples, but each sample has a unique *S*_*0,0*_ value to be estimated. To assess the accuracy of this method with the data sets analyzed here all information on initial target concentration is ignored and the estimated S_0,0_ values for each sample is then compared to the known values.

### Separate curves method (Method D)

This method does not assume any similarity of rate constants among any of the samples and does not analyze samples simultaneously as methods A, B, and C do. Each sample is analyzed separately with all parameters estimated independently for each qPCR curve. Here the accuracy of method C is assessed by comparing the actual *S*_*0,0*_ values to the estimated ones. This is the type of analysis that is conventionally done when analyzing qPCR curves.

## Methods

### Data sets

Both models 1 and 2 were fit to two different data sets for which the actual initial concentrations of target are known. The model of Boggy and Woolf (2010) [14] was also fit to the same data sets and is called Model 0. The data sets used are: 1) Data of Boggy and Woolf (2010) [14], 2) Data of Rutledge (2004) [15] and are referred to as data set 1 and 2, respectively. The data of Boggy and Woolf (2010) [14] are for a 129 base pair synthetic target sequence with the following conditions for PCR: reaction vol = 25 μl, primer concentration = 400 nM each, detection system is SYTO-13 dye with concentration = 2 μM, dNTP concentration = 0.2 mM each. Six different absolute target concentrations range from 5 × 10^3^ to 5 × 10^8^ molecules per reaction volume at 10 fold increments. The data of Rutledge (2004) [15] is for the 102 base pair K1/K2 target sequence with the following conditions for PCR: reaction vol = 35 μl, primer concentration = 0.25 μM each, detection system is Syber Green dye. Six different absolute target concentrations ranged from 4.17 × 10^2^ to 4.17 × 10^7^ molecules per reaction volume at 10 fold increments. Data set 1 has 2 replicate qPCR curves for each target concentration and data set 2 has 5 replicates for each target concentration.

### Determining baseline fluorescence

Before fitting a model the baseline fluorescence was determined for each qPCR curve by fitting the sigmoidal function given in equation 8 below.

(8) $$F_{n}{=}_{b}F+\frac{F_{\max}{-}_{b}F}{1+\exp\left\{-\frac{n-c_{1/2}}{k_{s}}\right\}}$$

The first term on the right side of equation 8 is the baseline fluorescence, _*b*_*F* . The second term is a modified sigmoidal function describing the increase in fluorescence due to polymerase chain reaction where *F*_*max*_ is the maximum fluorescence, *c*_*1/2*_ is the cycle number at which *F*_*n*_ is midway between _*b*_*F* and *F*_*max*_*,* and *k*_*s*_ is a scale parameter. Parameters in equation 8 and bounds used in the non-linear estimation program are _*b*_*F* ≥ 0, *F*_*max*_ > _*b*_*F* , c_1/2_ > 1, and *k*_*s*_ > 0. Equation 8 is fit to each entire qPCR curve by nonlinear estimation of the parameters using least squares as the criterion for fit with the PROC NLIN procedure of the SAS program version 9.3 [16]. Each observation was weighted by the reciprocal of the observed fluorescence. The value of _*b*_*F* is estimated separately for each qPCR curve and then treated as a constant for that curve when estimating parameters of models 0, 1, and 2. In preliminary analyses for this study the baseline fluorescence was estimated as a parameter in the model with each qPCR curve having its own unique value. In the resulting fits some qPCR curves were found to have baseline values that were not consistent with the observed values in the lag phase. This is due to the estimation procedure selecting a baseline value that gives the best overall fit to the curve rather than just in the lag phase. Ruijter *et. al.* (2009) [9] have shown that baseline estimates can affect the quality of fit in the exponential phase for the C_t_ method and this same effect is likely occurring in fits of the models here. They also point out inherent problems with estimating baseline fluorescence from a fixed number of points in early cycles. Rutledge and Stewart (2008) [1] showed sigmoidal functions similar to equation 8 give very good fit to the observed values of qPCR curves. The use of the sigmoidal function given in equation 8 is found to always give values that fit well to data in the lag phase and is not affected much by peculiar changes in fluorescence often seen during the first few cycles.

### Estimation of model parameters

Methods described here are used to determine how well each model explains the shape of each qPCR curve and also to determine how accurately each model estimates initial target concentration for each of the two data sets. Each model, 0, 1 or 2, was fit to qPCR data by nonlinear curve fitting with PROC NLIN of SAS version 9.3 [16] using the Marquardt optimization method and the mean sum of squared differences between the observed and predicted fluorescence values (MSresidual) as the measure of goodness-of-fit. Mean Square Residual (MSresidual) is a measure of how well a model explains the qPCR curve. A MSresidual value near zero indicates a very good fit in which the model predicted values are all very close to the observed values and higher values indicate a poorer fit. Up to 10,000 iterations were used for each fit. The parameters estimated and bounds imposed during estimation are: *S*_*0,0*_ > 0, k > 0 for model 0, *S*_*0,0*_ > 0 , *K*_*f*_ >0, *K*_*s*_ > *K*_*D12*_ >0 for model 1, and *S*_*0,0*_ >0 , *K*_*f*_ >0 , and *K* >0 for model 2. The initial concentrations of primers were set to the values reported by the authors providing the two data sets and expressed as nmoles/L. Additional file 4 contains SAS code that fits model 1 and 2 to a sample data set.

### Effect of varying L

To determine the effect of the choice of the value of L on the results of the analysis, the entire analysis was done separately for each of a range of values for L. Specifically, each of the three models (0, 1, 2) was fit using each of the estimation methods (A, B, C, D) to each of the two data sets (1, 2) for each of the L values of 0.1, 0.2, 0.3, 0.4, 0.5, 0.6, 0.7, 0.8, 0.9, 0.95, 0.98. The MSresidual and Fold Error for each fit were averaged over replicates 1 and 2 for each data set and the resulting means plotted versus L for each dilution separately.

### Effect of errors in estimation of baseline fluorescence

Analyses were done to assess the effect of errors in the estimation of baseline fluorescence, _b_F, on the goodness of fit of the model and the estimated initial target concentration. The estimation procedure used to estimate _b_F provides the standard error of the estimated value. Fits of the models and estimation of initial target concentration was done using _b_F values 1 and 2 standard deviation units above and below the estimated value. Plots of the mean MSresidual and Fold Error in estimation of initial target concentration versus the _b_F value used were used to assess the effect of errors in estimation of _b_F.

## Results

**Figure 4:**
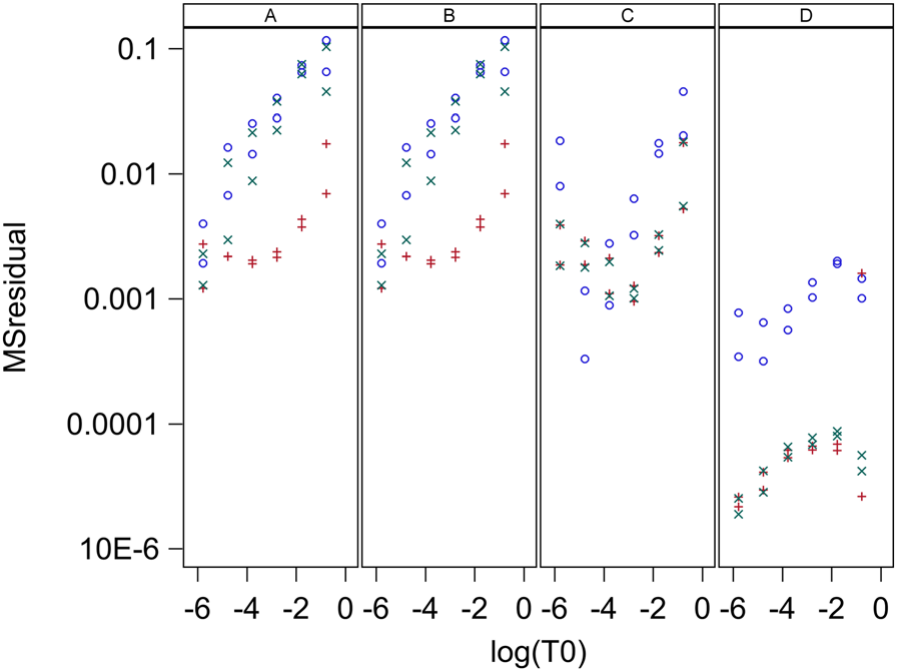
**Goodness of fit to qPCR curves for data set 1 with L = 0.8.** Plots are MS_residual_ vs known initial log_10_ target concentration, T_0_, in nM/L. Panels A,B,C and D contain plots for the Standard curve, Dilution curve, Simultaneous curves, and Separate curves estimation methods, respectively. Plot symbols are: blue circle = model 0, red plus = model 1, green X = model 2.

**Figure 5:**
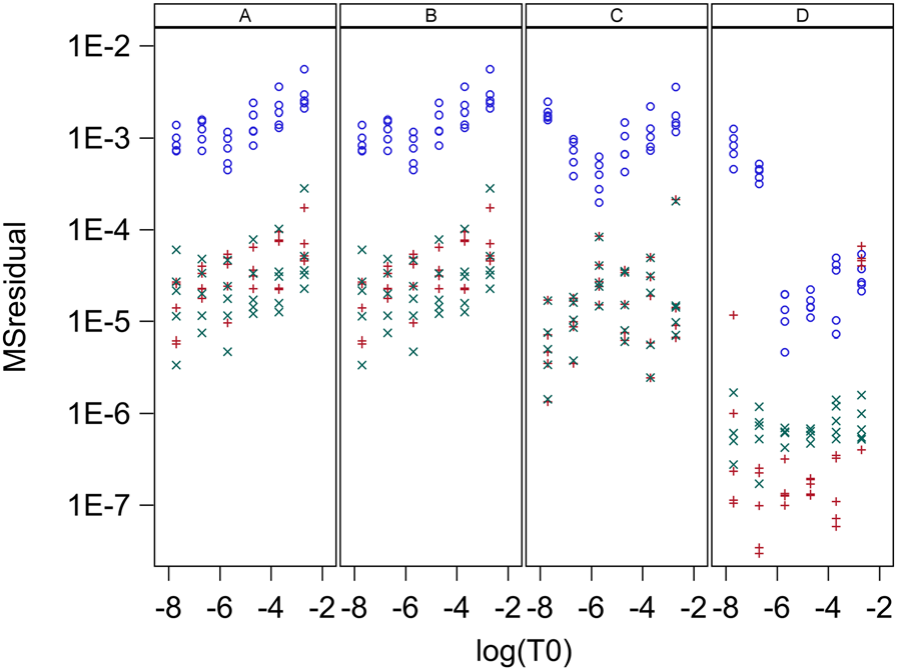
**Goodness of fit to qPCR curves for data set 2 with L = 0.8.** Plots are MS_residual_ vs known initial log_10_ target concentration, T_0_, in nM/L. Panels A,B,C and D contain plots for the Standard curve, Dilution curve, Simultaneous curves, and Separate curves estimation methods, respectively. Plot symbols are: blue circle = model 0, red plus = model 1, green X = model 2.

**Figure 6:**
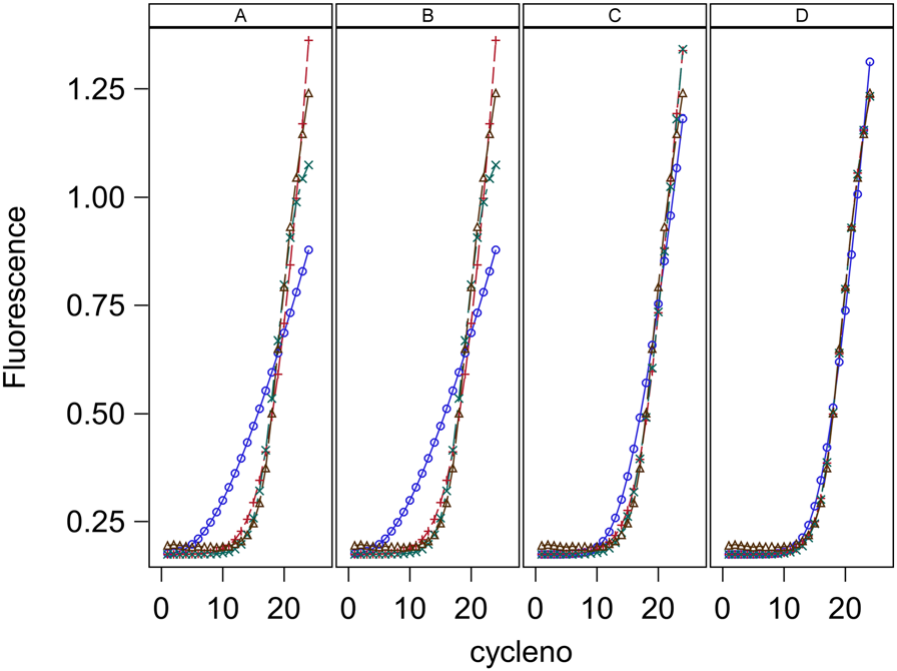
**Fit of models to qPCR curves for each model and each estimation method for data set 1, rep = 1, dilution = 1E-2, and L = 0.8.** Plot symbols for predicted fluorescence are:blue circle = model 0, red plus = model 1, green X = model 2. The plot symbol for observed fluorescence is delta (**δ**).

For each of the three models (0,1,2), each of four methods for parameter estimation were used for each data set. Here every model and estimation method was used to fit the same data and MSresidual is used to compare models and methods in their goodness of fit to the data. A value of MSresidual was computed for every qPCR curve for each model and for each estimation method. Plots of MSresidual versus log_10_(T_0_) are given in Figure 4 for data set 1 and Figure 5 for data set 2. Data set 1 has two replicate MSresidual values for each target concentration and data set 2 has 5. These figures show that for both data sets and for all 4 methods of estimation, models 1 and 2 are very similar in fit to qPCR curves and both give better fit than model 0. The poorer fit for model 0 is always due to the predicted fluorescence being too high for low cycle numbers and too low for high cycle numbers. This pattern of lack of fit for model 0 is shown in Figure 6 which shows the fits for each model and estimation method for the first replicate of the sample with 5*10^6^ molecules in data set 1 and is representative of the fit found for all samples in both data sets. Note that in Figure 6, Models 1 and 2 fit the data well for all estimation methods (A,B,C,D) while Model 0 gives poor fit with methods A and B, moderately good fit with method C, and good fit only with method D.

### Estimation of absolute target concentration

**Figure 7:**
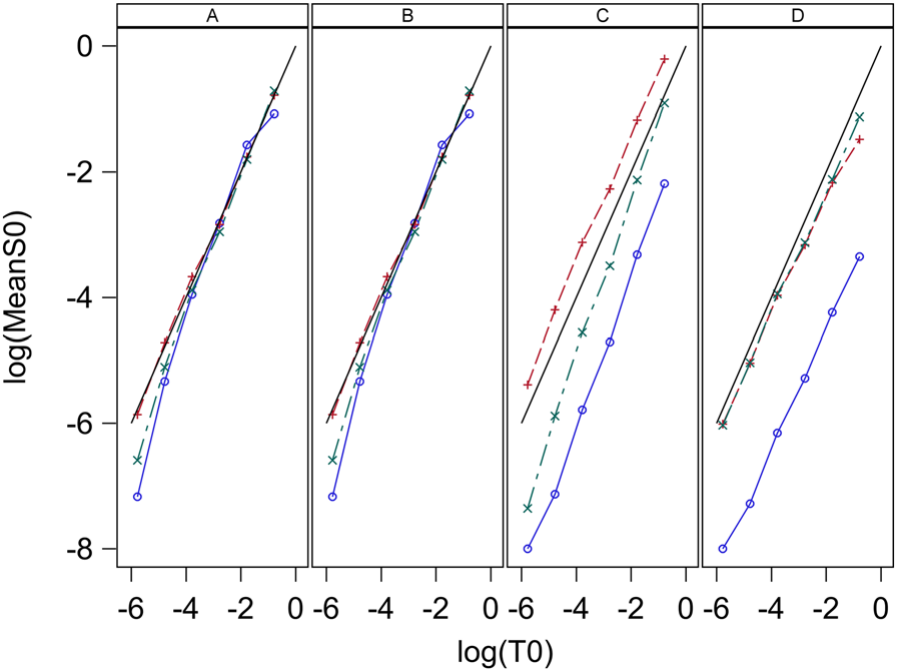
**Relation between log**_**10**_**(S**_**0**_**_Mean) and log**_**10**_**(T**_**0**_**) for analysis of data set 1 with L = 0.8.** log10S_0__Mean denotes base 10 log of mean of S_0_ for the two replicates and the solid line denotes log_10_(S_0__Mean) = log_10_(T_0_) which indicates perfect estimation. Panels A,B,C and D contain plots for the Standard curve, Dilution curve, Simultaneous curves, and Separate curves estimation methods, respectively. Plot symbols are: blue circle = model 0, red plus = model 1, green X = model 2.

**Figure 8:**
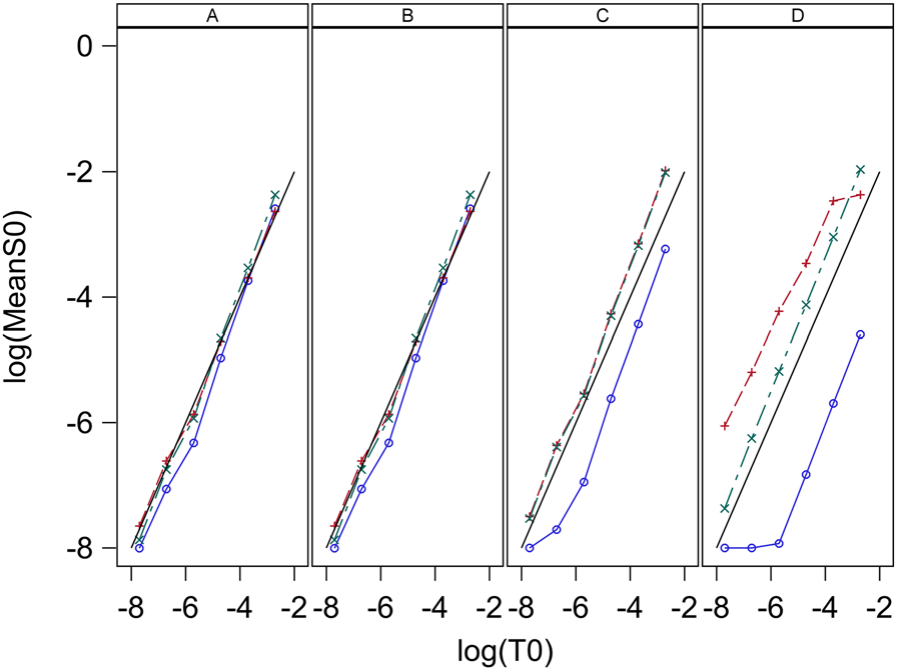
**Relation between log**_**10**_**(S**_**0**_**) and log**_**10**_**(T**_**0**_**) for analysis of data set 2 with L = 0.8.** log10S_0__Mean denotes base 10 log of mean of S_0_ for the 5 replicates and the solid line denotes log_10_(S_0__Mean) = log_10_(T_0_) which indicates perfect estimation. Panels A,B,C and D contain plots for the Standard curve, Dilution curve, Simultaneous curves, and Separate curves estimation methods, respectively. Plot symbols are: blue circle = model 0, red plus = model 1, green X = model 2.

**Figure 9:**
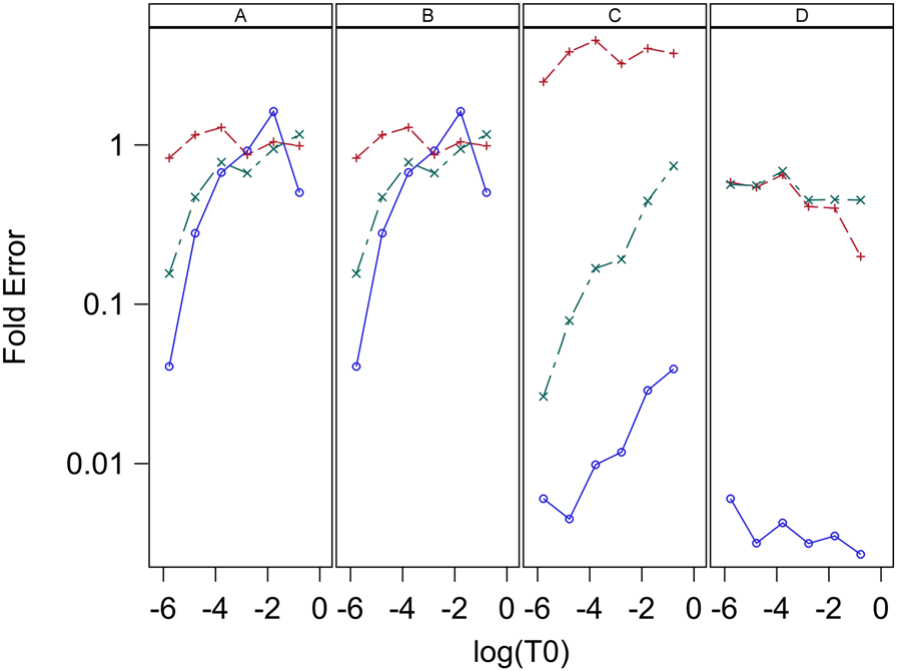
**Relation between Fold Error and log**_**10**_**(T**_**0**_**) for analysis of data set 1 with L = 0.8.** Fold Error is the ratio S0/T0 and a value of one indicates perfect estimation. Panels A,B,C and D contain plots for the Standard curve, Dilution curve, Simultaneous curves, and Separate curves estimation methods, respectively. Plot symbols are: blue circle = model 0, red plus = model 1, green X = model 2.

**Figure 10:**
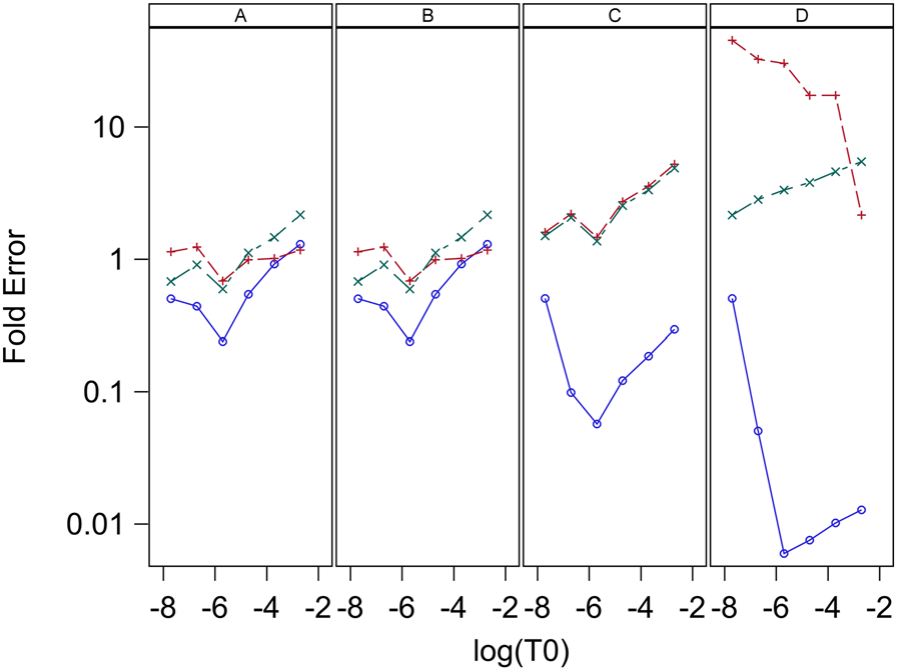
**Relation between Fold Error and log**_**10**_**(T**_**0**_**) for analysis of data set 2 with L = 0.8.** Fold Error is the ratio S0/T0 and a value of one indicates perfect estimation. Panels A,B,C and D contain plots for the Standard curve, Dilution curve, Simultaneous curves, and Separate curves estimation methods, respectively. Plot symbols are: blue circle = model 0, red plus = model 1, green X = model 2.

The estimates of initial target concentrations and the known initial target concentrations are denoted S_0,0_ and T_0_, respectively. Plots of log_10_(mean S_0,0_) vs log_10_(T_0_) for each model and estimation method are given in Figure 7 for data set 1 and Figure 8 for data set 2. Here means are over replicates for each sample. Figures 7 and 8 indicate all three models give S_0,0_ values that have a good linear relation to the true values, T_0_, for estimation methods A and B with both data sets. For estimation methods C and D the linear relation between S_0,0_ and T_0_ holds well for all three models when applied to data set 1 ,however, for data set 2 model 1 and 2 are very linear, but model 0 gives non-linear results. The high linearity of these log-log plots is not a good indication of the accuracy of the estimation because the estimates can be linear but at the same time be biased. A measure of the accuracy of the estimation is the Fold Error, which is the ratio of the estimated value to the true value (S_0_/T_0_). Fold error is computed for each model and estimation method and given in Figure 9 for data set 1 and Figure 10 for data set 2. Figures 9 and 10 show that for both data sets estimation methods A and B give similar results and model 1 gives more accurate estimation of T_0_ than the other two models. Estimation method C generally gave less accurate estimates than methods A and B and model 2 performed best. When using estimation method D, again model 2 gave the most accurate estimates over both data sets and model 0 was the least accurate for both data sets.

### Effect of varying L

**Figure 11:**
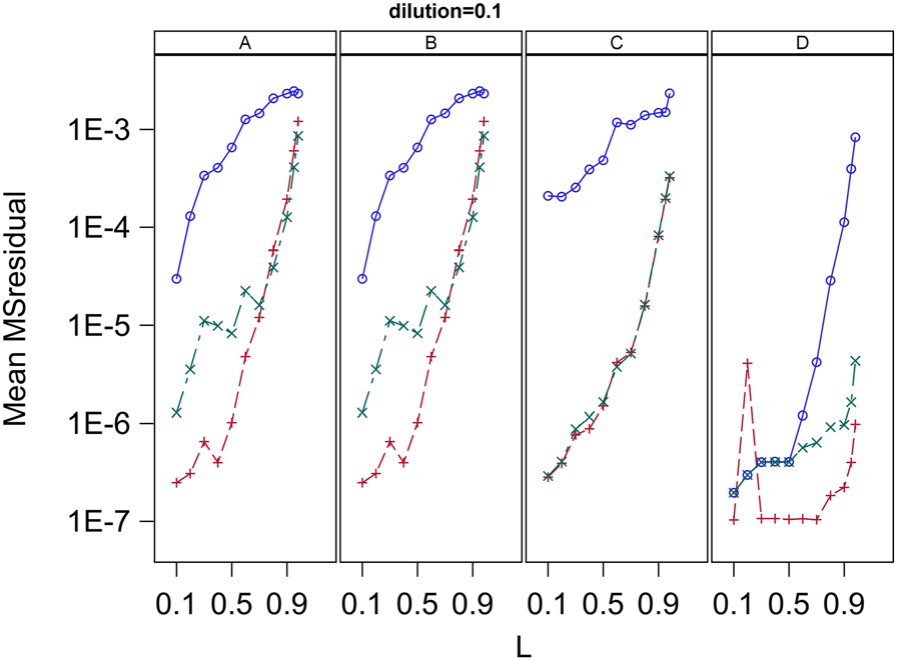
**Goodness of fit to qPCR curves for analysis of data set 2 with a range of L values.** Plots are mean MS_residual_ vs the value of L used in the analysis. Panels A,B,C and D contain plots for the Standard curve, Dilution curve, Simultaneous curves, and Separate curves estimation methods, respectively. Plot symbols are: blue circle = model 0, red plus = model 1, green X = model 2.

**Figure 12:**
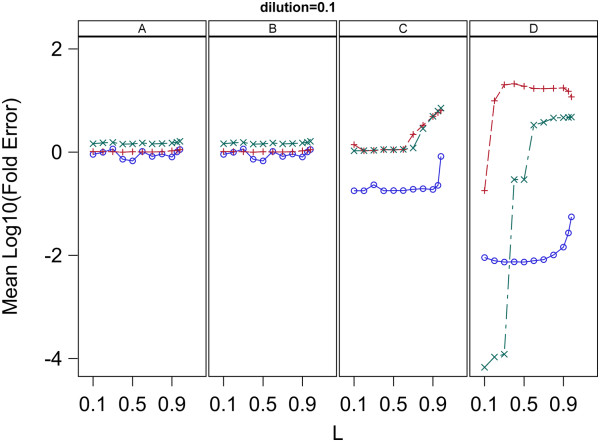
**Relation between log**_**10**_**(Fold Error) and L for analysis of data set 2 with a range of L values.** log10S_0__Mean denotes base 10 log of mean of S_0_ for the replicates. A value of 0 for log_10_(Fold Error) indicates perfect estimation. Panels A,B,C and D contain plots for the Standard curve, Dilution curve, Simultaneous curves, and Separate curves estimation methods, respectively. Plot symbols are: blue circle = model 0, red plus = model 1, green X = model 2.

The plots of the Mean MS residual vs L and Mean log(Fold Error) vs L for the samples with dilution factor 0.1 in data set 2 are given in Figures 11 and 12, respectively. This plot as well as plots for all dilutions in both data set 1 and 2 are given in Additional file 5. The plots given in Figures 11 and 12 were chosen because they show patterns which are representative of most of the plots given in Additional file 5.

### Effect of errors in estimation of baseline fluorescence

Plots of mean MSresidual and fold error in estimated initial target concentration versus baseline fluorescence for estimation methods A, B, and D are affected very little by variation in the value of _b_F used (plots not reported here). When using estimation method C, the MSresidual values were not affected much by variations in the _b_F but the initial target concentrations were over-estimated when using low values of _b_F. Increases in Fold Error by a factor of up to 10 was found in some plots when a _b_F value two standard error units below the estimated value was used.

## Discussion

### Fit to qPCR curves

The two models presented here have both been shown to give good fit to the lag and exponential phase of qPCR curves over a wide range of initial target concentrations while using a single set of rate constants. The lack of fit of model 0 to qPCR curves seen in Figures 4, 5 and 6 is likely to be due to the same cause as the variation in k values pointed out by Boggy and Woolf (2010) [14] . The derivation of the Boggy and Woolf [14] formula assumes their parameter k = k_a_/k_a12_ where k_a_ and k_a12_ are the rate constants of model 2 (Figure 3). Thus the same value of k should apply to all qPCR curves that are identical except for initial target concentration. I have shown that the parameter k of the Boggy and Woolf (2010) [14] model is not constant but depends on the primer concentration during the PCR process. Estimation methods A, B, and C force model 0 to use the same k value for all curves and thus each curve gives a poorer fit than it would if allowed to have a unique k value for each curve. These results suggest the Boggy and Woolf (2010) [14] assumption that primer concentration is sufficiently large that it can be ignored is not met in these data sets.

### Estimation of T_0_

The results here indicate the best methods for estimation of target concentration in an unknown sample is to use a standard curve that has a range of values for initial target concentration or to use a dilution curve composed of a series of dilutions of the original sample and analyze the qPCR curve with estimation methods A and B, respectively. Figures 4, 5, 7, 8, 9, and 10 indicate these two methods (A and B) are essentially equivalent in their accuracy of estimation. Note that with the dilution curve method, B, the absolute initial concentrations of target are estimated independently of the known values. Only the dilution ratios are used in the simultaneous fit to the data. The best way to estimate target concentration of an unknown sample when a standard curve is not available is to make a series of dilutions of the sample and then apply qPCR to each dilution and use the dilution curve method described here to estimate the target concentration of the undiluted sample. The separate curves method, D, worked well for data set 1 for both models 1 and 2, and worked moderately well only for model 1 for data set 2. Lastly, estimation method C gave good estimates for data set 2 but poorer estimates for data set 1. More sources of data need to be studied to determine the suitability of estimation methods C and D with these models.

The poor fit of the Boggy and Woolf (2010) [14] model to qPCR curves with estimation methods A, B, and C suggest it will give poorer estimates of initial target concentration than models giving good fit. Estimation method D allows each curve to have unique parameter values and gives much better fit to qPCR curves when using model 0, however, the accuracy of estimates is not as good as those for models 1 and 2. The non-linearity of initial target concentration estimates for Model 0 with method D using data set 2 as seen in Figure 8 panels C and D data is different than the result reported by Boggy and Woolf (2010) [14] who obtained a very linear relationship using method D. The reason for this difference is not clear but may be due to the fact that model fitting is done differently. Boggy and Woolf (2010) [14] estimated baseline fluorescence as a parameter in the model. I estimate baseline fluorescence before fitting the model and treat it as a constant during model fitting. Other minor differences in the programs used for fitting and the searching routine may have also caused some difference in the result.

### Effect of varying L

Figures 11 and 12 as well as those given in Additional file 5 indicate the effects of variation of L depend on the estimation method used and to a lesser extent on the concentration of initial target sequence. Examination of the plots in Additional file 5 indicate the effects of L can be variable but some general patterns are present which are shown in Figures 11 and 12. First, for all models, all methods and all dilutions, MSresidual increases with increasing L. All the plots involve a log scale and show an approximate linear increase of MSresidual with L indicating an underlying exponential increase of MSresidual with L. Thus when L has values of 0.9 and higher the fits to qPCR curves are much worse than with lower values of L. Secondly, the effect of L on the accuracy of the estimated initial target concentration depends strongly on the estimation method used. Estimated target concentrations obtained with estimation methods A and B show remarkably little dependence on the value of L used for either of the data sets analyzed. There is a consistent trend for a slight over-estimation of target concentration for L values above 0.8 with the amount of over-estimation increasing with increasing L. The dependency of the accuracy of estimation by methods C and D on L was more variable, however, both methods showed a patterns similar to that present in methods A and B, but more extreme over-estimation of initial target concentration when L values above 0.8 are used. For these reasons the value of 0.8 was used for L in the analyses presented here. Though the use of L = 0.8 gave good fits to qPCR curves and accurate estimates of initial target concentration, investigation of other methods for restricting analysis to only parts of the qPCR curve may be worthwhile.

### Effect of errors in estimation of baseline fluorescence

Estimation methods A, B and D are robust to errors in baseline fluorescence. Estimation method C is less robust to such errors.

### General comments

The models presented here assume the annealing of single-stranded DNA to double-stranded molecules reaches equilibrium at each cycle. This assumption may not be true, however, the ability of the present models to fit qPCR curves as well as they do suggests it may be approximately true. Model 1 gave slightly better fits to qPCR curves and slightly better estimates of initial concentration than Model 2 when estimation methods A and B were used. This result suggests the reversible reactions assumed in model 1 (Figure 2) may be a better model than the non-reversible reactions assumed in model 2 (Figure 3). It is thought the dissociation constant for double-stranded templates is so small that the annealing of templates is effectively non-reversible [8]. However, the annealing of primer and template may produce a much less stable double-stranded structure such that the reversible reaction model is more reasonable. A model in which template-template molecules are completely stable and primer-template molecules are not is not useful when considering equilibrium solutions, as equilibrium then occurs only when the concentration of primer-template molecule is zero. To use such a model, a non-equilibrium solution would need to be found. It is also possible that there is not enough time allowed during the experiments for the re-association reactions to reach equilibrium. Whether or not DNA re-association achieves equilibrium or not during PCR is not clear. Smith *et. al.* (2007) [13] offer some analysis suggesting that equilibrium does occur.

An advantage of the mechanistic approach to modeling qPCR curves is that models describe actual events of the process and thus may be expanded to include other effects that may improve accuracy. Kinetic models naturally allow simultaneous estimates of common parameters which will increase accuracy. This is extremely difficult or impossible with other methods used for modeling qPCR. Because the kinetic models account for variable efficiency by kinetic theory they may give better estimates of initial target concentration than other approaches. Another advantage of kinetic models is that parameters of the model may be estimated by dedicated experiments distinct from the qPCR curve experiment. For example, the parameter *K*_*f*_ present in both models 1 and 2 converts double-stranded DNA concentration into amount of fluorescence for a particular experimental system. The value of *K*_*f*_ could be determined in experiments separate from qPCR and then used as a constant in the analysis of a qPCR curve, thus increasing the accuracy of estimation of the kinetic parameters and target concentrations. Lastly, kinetic models uniquely allow estimation of absolute initial concentrations of target sequence without use of a standard curve of any type. The accuracy of estimation with kinetic models is enhanced greatly by the use of a standard curve, but it is not required. In fact the dilution curve method gave fits essentially as good as the standard curve method. The mechanistic models developed here offer a more complete description of the amplification occurring in qPCR, fit observed data very well, and allow more accurate estimation of initial target concentration than other methods.

## Conclusions

Two stepwise kinetic equilibrium models of qPCR are presented and analytical solutions are given for equilibrium values during annealing of single stranded to double stranded molecules. The models are amenable to different types of non-linear fitting which include fitting several curves simultaneously when they have common parameter values. Both models are shown to give very good fit to qPCR data with a wide range of initial target concentrations with a single set of values for rate constants. The two models also give accurate estimates of initial absolute target concentration using several different methods for estimation. Using the models with data from a standard curve gives accurate estimates of initial absolute target concentration. In the absence of a standard curve, a dilution curve method also provided accurate estimates of the initial absolute target concentration. In the absence of either a standard or dilution curve the models provide estimates, though less accurate, of absolute initial target concentration. These models presently give the best unified theory for the interpretation of qPCR data in that they explain well the shape of the qPCR curve and how it is affected by variation in the initial target concentration.

## Competing interests

The authors declare that they have no competing interests.

## Authors’ contributions

Gary Cobbs did the entire project.

## Supplementary Material

Additional file 1 Finding equilibrium for Model 1. Click here for file

Additional file 2 Finding equilibrium for Model 2. Click here for file

Additional file 3 Relating Model 2 to the results of Boggy and Woolf (2010) [14]. Click here for file

Additional file 4 SAS commands for Models 1 and 2. Click here for file

Additional file 5 Effect of varying L on MSresidual and Fold Error. Click here for file
