# Supplementary material for: Stepwise kinetic equilibrium models of quantitative polymerase chain reaction
Source: BMC Bioinformatics. 2012 Aug 16;13:203. doi: 10.1186/1471-2105-13-203 (PMC3519511; doi:10.1186/1471-2105-13-203)
Supplement: Additional file 1 — Finding equilibrium for Model 1. [file 1471-2105-13-203-S1.pdf]

Additional File 1: Finding equilibrium for Model 1

Setting equations in Figure 2B equal to zero gives

$$0 = -k_a P_{n,e} S_{n,e} - k_{a12} S_{n,e}^2 + k_d Q_{n,e} + k_{d12} D_{n,e} \quad \text{(A1.1a)}$$

$$0 = -k_a P_{n,e} S_{n,e} + k_d Q_{n,e} \quad \text{(A1.1b)}$$

$$0 = k_a P_e \cdot S_e - k_d Q_e \quad \text{(A1.1c)}$$

$$0 = k_{a12} S_{n,e}^2 - k_{d12} D_e \quad \text{(A1.1d)}$$

Rearranging equations A1.1b and A1.1d gives

$$P_{n,e} S_{n,e} = K_s Q_{n,e} \quad \text{(A1.2a)}$$

$$S_{n,e}^2 = K_D D_{n,e} \quad \text{(A1.2b)}$$

where $K_s = k_d/k_a$ and $K_{D12} = k_{d12}/k_{a12}$.

Conservation of total amount of primer sequence whether incorporated into products or not and conservation of total target whether single- or double-stranded gives equations A1.3a and A1.3b, respectively, below.

$$P_{n,0} = P_{n,e} + Q_{n,e} \qquad \text{(A1.3a)}$$

$$S_{n,0} = S_{n,e} + Q_{n,e} + D_{n,e} \qquad \text{(A1.3b)}$$

Rearranging equation A1.3a gives equation A1.4a below and substituting equation A1.3a into equation A1.3b gives equation A1.4b below.

$$Q_{n,e} = P_{n,0} - P_{n,e} \qquad \text{(A1.4a)}$$

$$D_{n,e} = S_{n,0} - S_{n,e} - \left(P_{n,0} - P_{n,e}\right) \qquad \text{(A1.4b)}$$

Substituting equation A1.4a into equation A1.2a and rearranging gives equation A1.5a below and substituting equation A1.4b into equation A1.2b gives equation A1.5b below.

$$P_{n,e} = P_{n,0}\left(\frac{K_s}{K_s + S_{n,e}}\right) \qquad \text{(A1.5a)}$$

$$S_e^2 = K_D\left\{S_{n,0} - S_{n,e} - \left(P_{n,0} - P_{n,e}\right)\right\} \qquad \text{(A1.5b)}$$

Substituting equation A1.5a into equation A1.5b gives

$$S_e^2 = K_D\left\{S_{n,0} - S_{n,e} - P_{n,0}\left(1 - \frac{K_s}{K_s + S_{n,e}}\right)\right\}$$

which upon rearrangement gives

$$S_{n,e}^{3} + \left(K_s + K_{D12}\right)S_{n,e}^{2} + K_D\left(P_{n,0} - S_{n,0} + K_s\right)S_{n,e} - K_s K_{D12} S_{n,0} = 0 \qquad \text{(A1.6)}$$

where $K_s = k_d/k_a$ and $K_D = k_{d12}/k_{a12}$. In equation A1.6, coefficients of the 2nd and 3rd degree terms are always positive, the coefficient of the 1st degree term may be positive, negative or zero, and the constant term is always negative. These conditions indicate equation A1.6 always has exactly one real positive root and this root is denoted $S_{n,e}$. Finding the real root, $S_{n,e}$, of eq A1.6 by the cubic equation [1] is shown below.

Define a = 1, b = $K_s + K_D$, c = $K_D(P_{n,0} - S_{n,0} + K_s)$, d = $K_s\, K_D\, S_{n,0}$.

Then define p = $\{3c/a - (b/a)^2\}/3$ and q = $\{2(b/a)^3 - 9bc/a/a + 27d/a\}/27$

and define $D = (p/3)^3 + (q/2)^2$.

if $D \geq 0$ then the roots of equation A1.6 are given by

$$S_{n,e} = \left\{\frac{-q}{2} + D^{\frac{1}{2}}\right\}^{\frac{1}{3}} + \left\{\frac{-q}{2} - D^{\frac{1}{2}}\right\}^{\frac{1}{3}} - b/a/3 \qquad \text{(A1.7a)}$$

if D < 0 then then the roots of equation A1.6 are given by

$$S_{n,e} = 2\left(\frac{|p|}{3}\right)\cos\left(\frac{\varphi}{3}\right) - b/a/3 \quad \text{where} \quad \varphi = a\cos\left\{\frac{\frac{-q}{2}}{\sqrt{\frac{|p|^3}{27}}}\right\} \qquad \text{(A1.7b)}$$

The relevant root of equation A1.6 is found using the cubic formula given in equation A1.7.

Equilibrium values for the remaining state variables may be found by substituting the value of

$S_{n,e}$ into equations A1.8, A1.9, and A1.10 below.

$$P_{n,e} = P_{n,0}\left(\frac{K_s}{K_s + S_{n,e}}\right) \qquad n = 1, 2, 3, \ldots \qquad \text{(A1.8)}$$

$$Q_{n,e} = P_{n,0}\left(\frac{S_{n,e}}{K_s + S_{n,e}}\right) \qquad n = 1, 2, 3, \ldots \qquad \text{(A1.9)}$$

$$D_{n,e} = \frac{S_{n,e}^2}{K_D} \qquad n = 1, 2, 3, \ldots \qquad \text{(A1.10)}$$

References

1. Wolfram MathWorld [http://mathworld.wolfram.com/CubicFormula.html]
