# Supplementary material for: Stepwise kinetic equilibrium models of quantitative polymerase chain reaction
Source: BMC Bioinformatics. 2012 Aug 16;13:203. doi: 10.1186/1471-2105-13-203 (PMC3519511; doi:10.1186/1471-2105-13-203)
Supplement: Additional file 2 — Finding equilibrium for Model 2. [file 1471-2105-13-203-S2.pdf]

Additional File 2: Finding equilibrium for Model 2

Start with equations in Figure 3B which are repeated below.

$$\frac{dS_n}{dt} = -k_a P_n S_n - k_{a12} S_n^2 \qquad S(0) = S_0 \qquad \text{(A2.1a)}$$

$$\frac{dP_n}{dt} = -k_a S_n P_n \qquad P(0) = P_0 \qquad \text{(A2.1b)}$$

Dividing equation A2.1a by equation A2.1b gives

$$\frac{dS_n}{dP_n} = (K_r)\left(\frac{S_n}{P_n}\right) + 1 \quad \text{where} \quad K_r = \frac{K_{a12}}{K_a} \quad . \qquad \text{(A2.2)}$$

Solution of separable first order ordinary differential equations of the form $\frac{dy}{dx} = F\left(\frac{y}{x}\right)$ may be found by integrating both sides of $\frac{dx}{x} = \frac{d\nu}{F(\nu) - \nu}$ where $\nu = \frac{y}{x}$ [1]. Defining $F(r_n) = (K_r)r_n + 1$ and $r_n = \frac{S_n}{P_n}$, then the solution of equation A2.2 may be obtained by integrating both sides of $\frac{dP_n}{P_n} = \frac{dr_n}{(K_r - 1)r_n + 1}$. Assuming $K_r > 1$ and $r_n \geq 0$, and

evaluating definite integrals with initial values $P_{n,0}$ and $r_{n,0}$, gives

$$\ln\left(P_{n,t}\right) - \ln\left(P_{n,0}\right) = \left(\frac{1}{K_r - 1}\right)\left[\ln\left\{\left(K_r - 1\right)r_{n,t} + 1\right\} - \ln\left\{\left(K_r - 1\right)r_{n,0} + 1\right\}\right]$$

where $P_{n,t}$ is primer concentration at time t, $r_{n,t} = \frac{S_{n,t}}{P_{n,t}}$ where $S_{n,t}$ is single-stranded template

concentration at time t. This rearranges to

$$P_{n,t} = P_{n,0}\left\{\frac{K + \left(\frac{S_{n,t}}{P_{n,t}}\right)}{K + \left(\frac{S_{n,0}}{P_{n,0}}\right)}\right\}^{K} \qquad \text{where } K = \frac{k_a}{k_{a12} - k_a} \qquad \text{(A2.3)}$$

Equation A2.3 is an implicit solution of equation A2.2. Equilibrium occurs when t → ∞ and at equilibrium $S_{n,\infty} = S_{n,e}$. Examination of equation A2.1a and eq A2.1b indicates $S_{n,\infty}=0$ is the only equilibrium solution for $S_{n,e}$. Substituting $S_{n,e} = 0$ into equation A2.3 when $t = \infty$ gives

$$P_{n,e} = P_{n,0}\left\{\frac{K}{K + \left(\frac{S_{n,0}}{P_{n,0}}\right)}\right\}^{K} \qquad \text{(A2.4)}$$

which is the equilibrium solution of equation A2.1. Conservation of total primer amount whether in single or double-stranded molecules gives $Q_{n,e} = P_{n,0} - P_{n,e}$ and using equation A2.4 gives

$$Q_{n,e} = P_{n,0}\left[1-\left\{\frac{K}{K+\left(\frac{S_{n,0}}{P_{n,0}}\right)}\right\}^{K}\right] \qquad \text{(A2.5)}$$

Conservation of total target amount, wether in single- or double-stranded molecules gives $S_{n,0} = S_{n,e} + Q_{n,e} + D_{n,e}$. Since $S_{n,e} = 0$ the equilibrium concentration of double-stranded target $D_{n,e} = S_{n,0} - Q_{n,e}$ and

$$D_{n,e} = S_{n,0} - P_{n,0}\left[1-\left\{\frac{K}{K+\left(\frac{S_{n,0}}{P_{n,0}}\right)}\right\}^{K}\right] \qquad \text{(A2.6)}$$

References

1. efunda [www.efunda.com/math/ode/ode1_separable.cfm.]
