# Supplementary material for: Stepwise kinetic equilibrium models of quantitative polymerase chain reaction
Source: BMC Bioinformatics. 2012 Aug 16;13:203. doi: 10.1186/1471-2105-13-203 (PMC3519511; doi:10.1186/1471-2105-13-203)
Supplement: Additional file 3 — Relating Model 2 to the results of Boggy and Woolf (2010) [14]. [file 1471-2105-13-203-S3.pdf]

Gary Cobbs: Stepwise Kinetic Equilibrium Models of qPCR Additional file 3

Relating Model 2 to the results of Boggy and Woolf (2010) [14]: The results presented by Boggy and Woolf (2010) [14] are obtained by assuming primer concentration is always much greater than target concentration and that variations in its concentration have no effect on the rate of production of primer-template duplex. This assumption led to their use of a simpler kinetic model in which the rate of conversion of template into primer-template duplex is first order in single-strand template concentration rather than second order as used here. They find equilibrium solutions to the simplified kinetic model which are then used to derive the recurrence relation given below.

$$D^*_{n+1} = D^*_n + k\ln\left(1 + \frac{D^*_n}{k}\right) \qquad \text{(A3.1)}$$

where D^* is the concentration of double stranded target DNA. The * is added to distinguish their quantity from mine. I show here that if the parameter $K = \frac{k_a}{k_{a12} - k_a}$ of model 2 is small my results for model 2 are equivalent to the result of Boggy and Woolf (2010) [14]. Let D^*_n denote the total concentration of double-stranded target molecules detected by the detection system. If all primers in target-primer duplexes are extended completely, then $D^*_{n+1} = 2Q_{n+1,e} + D_{n+1,e}$ and substituting this in equations A2.5 and A2.6 gives

$$D^*_{n+1} = S_{n+1,0} + P_{n+1,0}\left\{1 - \left(\frac{K}{K + \frac{S_{n+1,0}}{P_{n+1,0}}}\right)^K\right\} \qquad \text{(A3.2)}$$

where $K = \frac{k_a}{k_{a12} - k_a}$ Now, $S_{n+1,0} = D^*_n$ and $P_{n+1,0} = P_{n,e}$ which when substituted into equation A3.2 above and rearranging gives a recurrence relation for model 2 as

$$D^*_{n+1} = D^*_n + P_{n,e}\left\{1 - \left(\frac{KP_{n,e}}{KP_{n,e} + D^*_n}\right)^K\right\} \qquad \text{(A3.3)}$$

where $P_{n,e}$ is given by equation A2.4. Now expressing $\left(\frac{KP_{n,e}}{KP_{n,e} + D^*_n}\right)^K$ as a Maclaurin series in D^*_n gives

$$\left(\frac{KP_{n,e}}{KP_{n,e} + D^*_n}\right)^K = 1 - K\left(\frac{D^*_n}{KP_{n,e}}\right) + \frac{1}{2!}K(K+1)\left(\frac{D^*_n}{KP_{n,e}}\right)^2 - \frac{1}{3!}K(K+1)(K+2)\left(\frac{D^*_n}{KP_{n,e}}\right)^3 + \; - \; + \; .$$

which if K is small gives

$$\left(\frac{KP_{n,e}}{KP_{n,e}+D_n^*}\right)^K \approx 1-K\left[+\left(\frac{D_n^*}{KP_{n,e}}\right)-\frac{1}{2}\left(\frac{D_n^*}{KP_{n,e}}\right)^2+\frac{1}{3}\left(\frac{D_n^*}{KP_{n,e}}\right)^3 - + - \ldots\right]$$

The infinite series in the brackets is equal to $\ln\left(1+\frac{D_n^*}{KP_{n,e}}\right)$ thus

$$\left(\frac{KP_{n,e}}{KP_{n,e}+D_n^*}\right)^K \approx 1-K\ln\left(1+\frac{D_n^*}{KP_{n,e}}\right)$$

and substituting into equation A3.3 gives

$$D_{n+1}^* = D_n^* + KP_{n,e}\ln\left(1+\frac{D_n^*}{KP_{n,e}}\right)$$

which is the Boggy and Woolf (2010) [13] recurrence

relation, given here as equation 5, with their parameter k set to $KP_{n,e}$. The Boggy and Woolf (2010) [14] model is thus a special case of the present results for model 2 when K is small. This result indicates the parameter k of Boggy and Woolf (2010) [14] is not constant but depends on the value of $P_{n,e}$ which varies over cycles within a single qPCR and between qPCR experiments.
