# Supplementary material for: Stepwise kinetic equilibrium models of quantitative polymerase chain reaction
Source: BMC Bioinformatics. 2012 Aug 16;13:203. doi: 10.1186/1471-2105-13-203 (PMC3519511; doi:10.1186/1471-2105-13-203)
Supplement: Additional file 4 — SAS commands for Models 1 and 2. [file 1471-2105-13-203-S4.pdf]

Gary Cobbs: Stepwise Kinetic Equilibrium Models of qPCR Additional file 4

Below are SAS commands that fit models 1 and 2 to sample data.

```
* The commands below fit model 1 and model 2 to a sample qPCR curve;
* The file is known to work with SAS version 9.3 TS Level 1M0;
data PCR;
 input cycleno F @@;
 datalines;
 1  0.2004     2  0.1973     3  0.1968     4  0.1968     5  0.1969
 6  0.1954     7  0.1984     8  0.2008     9  0.2060    10  0.2177
11  0.2375    12  0.2807    13  0.3558    14  0.4836    15  0.6783
16  0.8919    17  1.0954    18  1.2747    19  1.4185    20  1.5416
21  1.6439    22  1.7195    23  1.7851    24  1.8313    25  1.8679
26  1.9056    27  1.9176    28  1.9352    29  1.9531    30  1.9511
31  1.9514    32  1.9596    33  1.9469    34  1.9394    35  1.9419
36  1.9276    37  1.9117    38  1.9084    39  1.8869    40  1.8781
;
proc print data=PCR;
 title1 'SAS code for fitting models 1 and 2 to sample qPCR data';
 title2 ' dataset PCR containing the original data on cycle number and fluorescence'; run;

*START OF LINES FOR Estimating baseline and adding it as variable Fb to dataset PCR;
ods listing select none;
proc nlin data=PCR method=marquardt maxiter=10000 noitprint;
 title2 ' Estimating baseline fluorescence, Fb, by fitting sigmoidal function';
 _weight_ = 1/F; * setting weights for fit;
 parameters Fb 0.1 0.2  Fmax 1.5 2.0  Chalf 10 15 20 25 30   ksigmoid  1; *starting values;
 bounds 0 < Fb; bounds 0<Fmax; bounds 0<Chalf; bounds 0<ksigmoid;  *setting bounds;
  do index=1 to cycleno;
    Fsig = Fb + (Fmax-Fb)/(  1  +  exp(-(cycleno-Chalf)/ksigmoid)   ) ;
  end; *for index;
 model F=Fsig;
 output out=nlinoutSigmoid predicted=pred parms= Fb Fmax Chalf ksigmoid; run;
ods listing select all;
data nlinoutSigmoid; set nlinoutSigmoid;
 keep cycleno F Fb Fmax;
 title3 '  dataset nlinoutSigmoid'; run;
proc sort data=PCR; by  cycleno;
proc sort data=nlinoutSigmoid; by cycleno;
data PCR; merge PCR nlinoutSigmoid; by cycleno; run;
proc print data=PCR;
 title2 ' dataset PCR after estimating baseline F = Fb and Fmax';  run;
* END OF LINES FOR Estimating baseline and adding it as variable Fb to dataset PCR;
proc plot data=PCR;
 title2 '  Plot of observed fluorescence, F, and background fluorescence, Fb';
 plot F*cycleno='o'  Fb*cycleno='b' / overlay; run;
data PCR; set PCR; L=0.8; if ((F-Fb)/(Fmax-Fb))>L then delete; run; *invoking limit L;
proc print data=PCR;
 title2 ' dataset PCR after removing cycles with fluorescence exceeding L'; run;

*  STARTING LINES FOR FITTING Model 1;
```

```
ods listing select none;
proc nlin data=PCR method=marquardt maxiter=10000 NOITPRINT;
 title2 ' Fitting model 1';
   parameters   Kf 1E-2 1E-1 1 1E1 1E2   Ks 1E-2 1E-1 1 10 50 100
                KD 1E-8 1E-6 1E-4 1E-2 1
                S0 1E-14 1E-13 1E-12 1E-11 1E-10 1E-9 1E-8 1E-7 1E-6 1E-5 1E-2;
   bounds 0 < Kf;  bounds 0 < Ks;  bounds 0 < KD;  bounds 0 < S0 ;
   PrimerConc=400;  P0=PrimerConc;   s0nminus1=S0; p0nminus1=P0;
   * Core program for Model 1 using Cubbic equation starts here, hat denotes eq values;
    do index=1 to cycleno;
     * Solution of cubic polynomial ax^3 + bx^2 +cx +d =0, by Cubic equation;
     a = 1;
     b = Ks+KD;
     c = -KD*(s0nminus1-P0nminus1-Ks);
     d = -KD*Ks*s0nminus1;
     p = ( 3*c/a - (b/a)**2)/3;
     q = ( 2*(b/a)**3 - 9*b*c/a/a + 27*d/a ) / 27;
     Disc = (p/3)**3 + (q/2)**2;
     *if Disc>=0 then;
     if Disc>=0 then u = sign(-q/2 +sqrt(Disc))*(abs(-q/2 +sqrt(Disc)))**(1/3);
     if Disc>=0 then v = sign(-q/2 -sqrt(Disc))*(abs(-q/2 -sqrt(Disc)))**(1/3);
     if Disc>=0 then y1real =  u + v;
     if Disc>=0 then y1imag =  0;
     if Disc>=0 then y2real = -(u+v)/2;
     if Disc>=0 then y2imag =  (u-v)*sqrt(3)/2;
     if Disc>=0 then y3real = -(u+v)/2;
     if Disc>=0 then y3imag = -(u-v)*sqrt(3)/2;
     if Disc>=0 then goto final;
     *if Disc<0 then;
     if Disc<0 then phi = arcos((-q/2)/sqrt((abs(p)**3)/27));
     if Disc<0 then y1real =  2*sqrt(abs(p)/3)*cos(phi/3);
     if Disc<0 then y1imag = 0;
     if Disc<0 then y2real = -2*sqrt(abs(p)/3)*cos((phi+constant('pi'))/3);
     if Disc<0 then y2imag = 0;
     if Disc<0 then y3real = -2*sqrt(abs(p)/3)*cos((phi-constant('pi'))/3);
     if Disc<0 then y3imag = 0;
     final:;
     x1real = y1real - b/a/3; x1imag = y1imag;
     x2real = y2real - b/a/3; x2imag = y2imag;
     x3real = y3real - b/a/3; x3imag = y3imag;
     Shatnminus1=x1real;
     *END OF Solution of cubic polynomial ax^3 + bx^2 +cx +d =0, by Cubic equation;
     Phatnminus1 = max(0, Ks*p0nminus1/(Ks+Shatnminus1));
     Qhatnminus1 = max(0, p0nminus1 - Phatnminus1);
     Dhatnminus1 = max(0, (s0nminus1 - Shatnminus1) - (p0nminus1 - Phatnminus1));
     s0n= s0nminus1 + Qhatnminus1;
        P0n= p0nminus1 - Qhatnminus1;
     E=(s0n/s0nminus1)-1; * E = efficiency of Amplification;

        *setting up values for next iteration;
     S0nminus1=S0n;
```

```
      POnminus1=P0-SOn+S0;
      FT= Kf*(2*Qhatnminus1 + Dhatnminus1);
     end; *for index;
     model F=Fb+FT;  *model using Fb as baseline;
    * END OF Core program for Model 1 using Cubic equation;
    ID sOn F E;
  output out=nlinout1 predicted=pred parms= Kf Ks KD S0; run;
 ods listing select all;
 proc print data=nlinout1;
  title2 ' dataset nlinout1'; run;
 proc plot data=nlinout1;
  title2 ' Plots of observed F and predicted F versus cyclenumber for Model 1';
  plot F*cycleno='F' pred*cycleno='p' / overlay; run;
 *  END OF LINES FOR FITTING Model 1;

 *  STARTING LINES FOR FITTING Model 2;
 ods listing select none;
 proc nlin data=PCR method=marquardt maxiter=10000 noitprint;
  title2 ' Fitting model 2';
    parameters   Kf  1E-1 1 1E1 1E2  K 1E-2 1E-1 1 1E1 1E2 1E3
                 S0 1E-9 1E-8 1E-7 1E-6 1E-5 1E-4;
    bounds 0 < Kf; bounds 1 < K; bounds 0 < S0 ;
    primerconc=400; *nmolar;
    P0=PrimerConc; sOnminus1=S0; pOnminus1=P0;
    * Core program for model 2 exact solution, hat denotes eq values;
     do index=1 to cycleno;
          *  Solving the DEQs by Exact formula;
    Phatnminus1 = POnminus1*((1/((K-1)*(sOnminus1/POnminus1)+1)))**(1/(K-1));
    Qhatnminus1 = POnminus1 - Phatnminus1;
    Dhatnminus1 = SOnminus1 - Qhatnminus1;
    Shatnminus1 = SOnminus1 - Dhatnminus1 - Qhatnminus1;
          *  END of Solving the DEQs by Exact formula;
      sOn= sOnminus1 + Qhatnminus1;
         POn= pOnminus1 - Qhatnminus1;
      E=(sOn/sOnminus1)-1; * E = efficiency of Amplification;
         *setting up values for next iteration;
      SOnminus1=SOn;
      POnminus1=P0-SOn+S0;
     end; *for index;
      FT= Kf*(2*Qhatnminus1 + Dhatnminus1);
     model F=Fb+FT;  *model using Fb as baseline;
    * END OF Core program for model 2 exact solution;
    ID sOn F E;
   output out=nlinout2 predicted=pred parms= Kf K S0; run;
 ods listing select all;
 proc print data=nlinout2;
  title2 ' dataset nlinout2'; run;
 proc plot data=nlinout2;
  title2 ' Plots of observed F and predicted F versus cyclenumber for Model 2';
  plot F*cycleno='F' pred*cycleno='p' / overlay; run;
 *  END OF LINES FOR FITTING Model 2;
```
