# Supplementary material for: Stepwise kinetic equilibrium models of quantitative polymerase chain reaction
Source: BMC Bioinformatics. 2012 Aug 16;13:203. doi: 10.1186/1471-2105-13-203 (PMC3519511; doi:10.1186/1471-2105-13-203)

Effect of varying L on MSresidual and Fold Error.

Each model (0,1,2) was fit to each replication (2 and 5 replications for datasets 1 and 2, respectively) for each dilution (1, 0.1, 0.01, 0.001, 0.0001, 0.00001) for each dataset (1,2) using each of the estimation methods (A, B, C, D) for each of eleven different values of L (L = 0.1, 0.2, 0.3, 0.4, 0.5, 0.6, 0.7, 0.8, 0.9, 0.95, 0.98). Thus there 2x6x4x11 = 528 fits for each model for dataset 1 and 5x6x4x11= 1320 fits for each model for dataset 2. The average MSresidual and average log10FoldError were computed by averaging over replicates for each dataset*dilution combination. Average MSresidual and average log10FoldError are plotted versus value of L for each dilution in each data set in Figures A4.1 - A4.? below.

Panels A,B,C and D are plots for the Standard curve, Dilution curve, Simultaneous curves, and Separate curves estimation methods, respectively. Plot symbols are: **o** = model 0, + = model 1, x = model 2.

Figure A4-1: Effect of varying L on MSresidual and Fold Error for Dataset 1 (Boggy and Woolf, 2010).

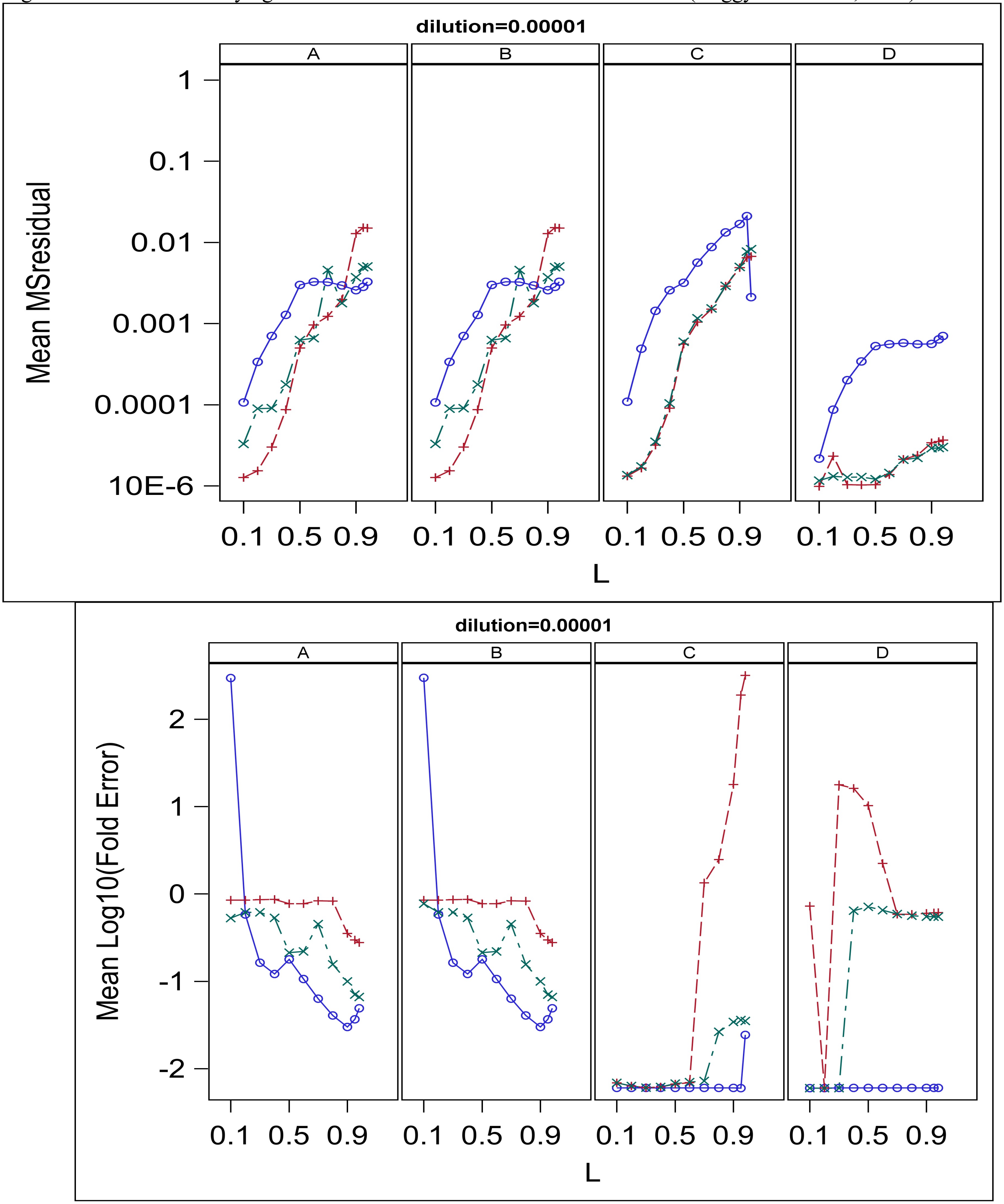

Figure A4-2: Effect of varying L on MSresidual and Fold Error for Dataset 1 (Boggy and Woolf, 2010).

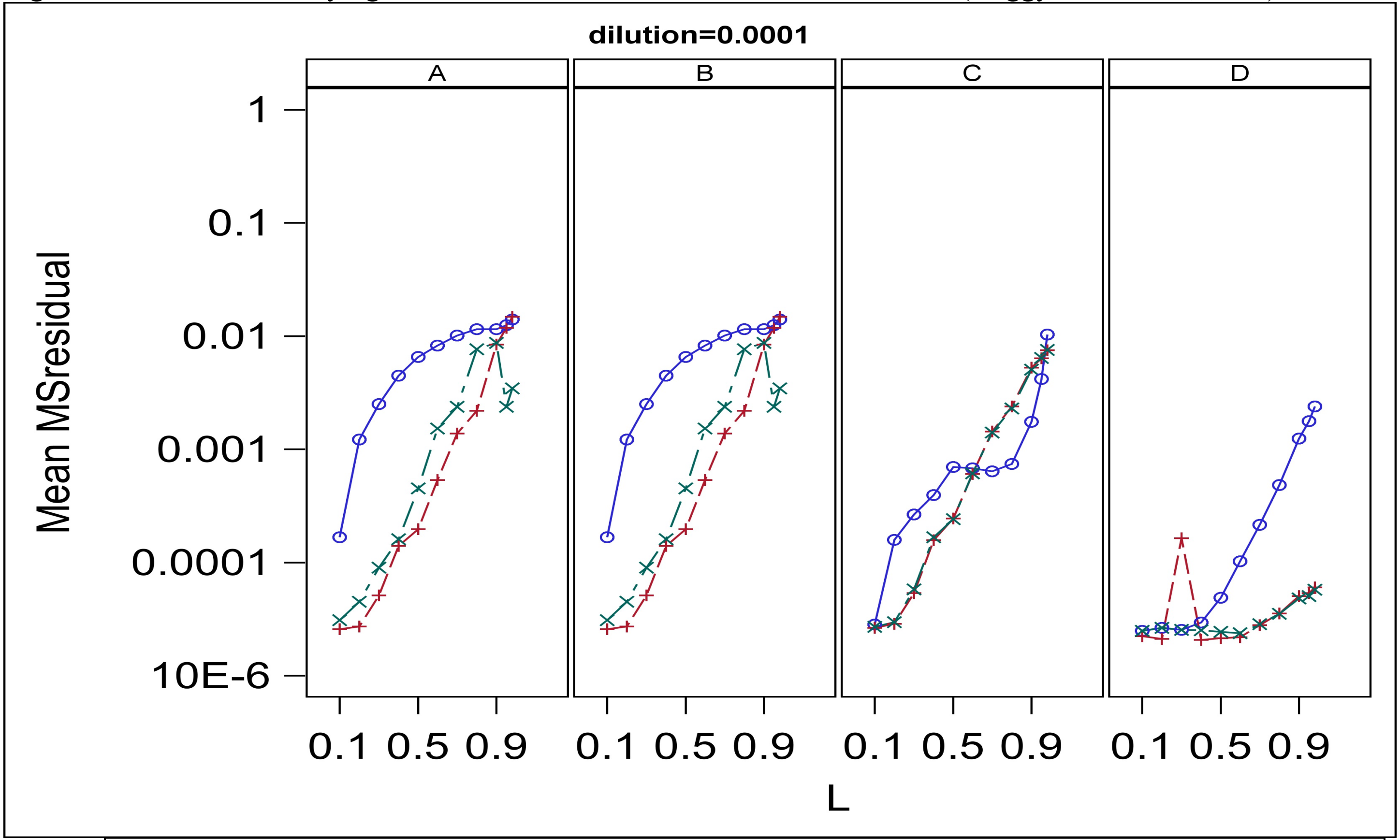

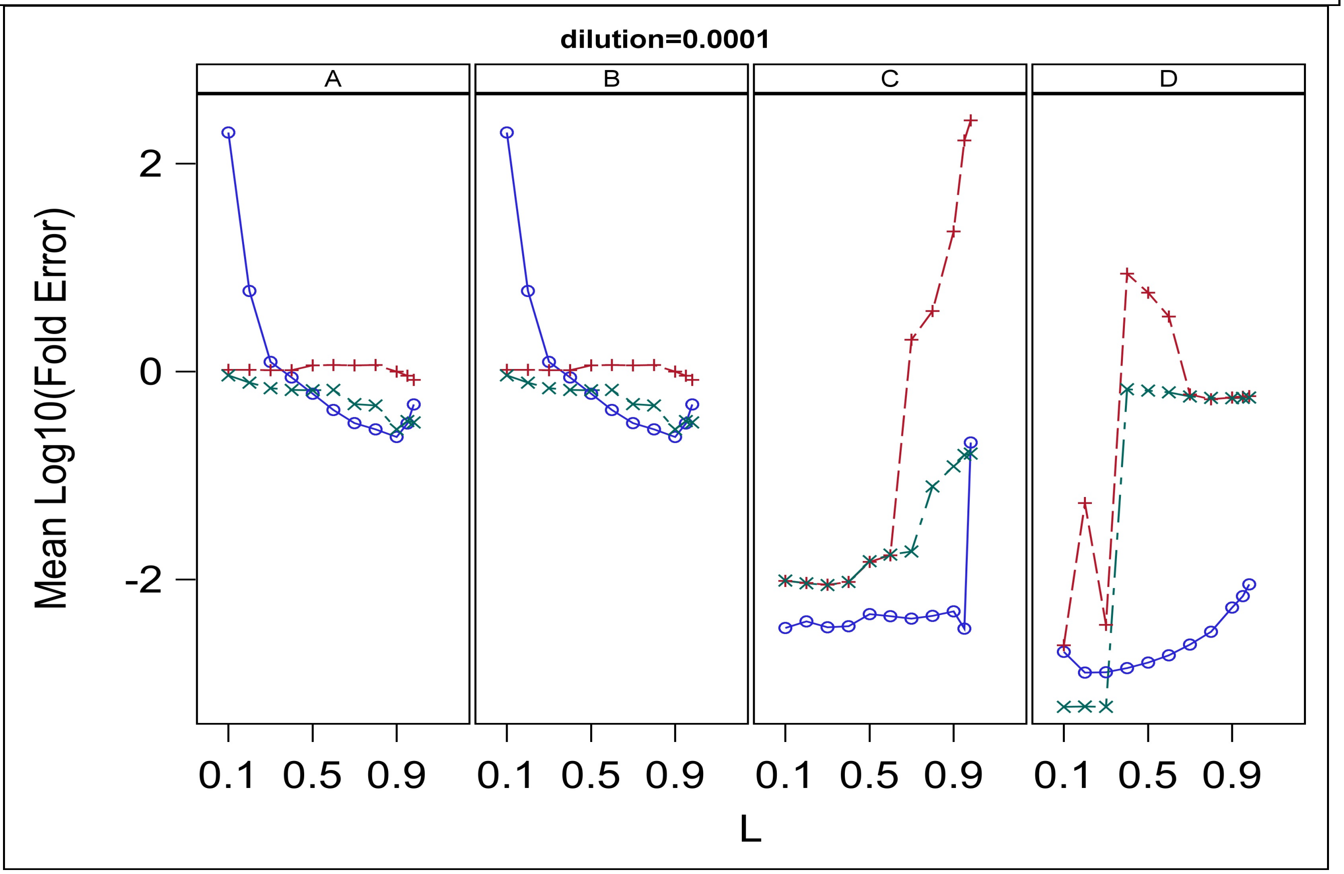

Figure A4-3: Effect of varying L on MSresidual and Fold Error for Dataset 1 (Boggy and Woolf, 2010).

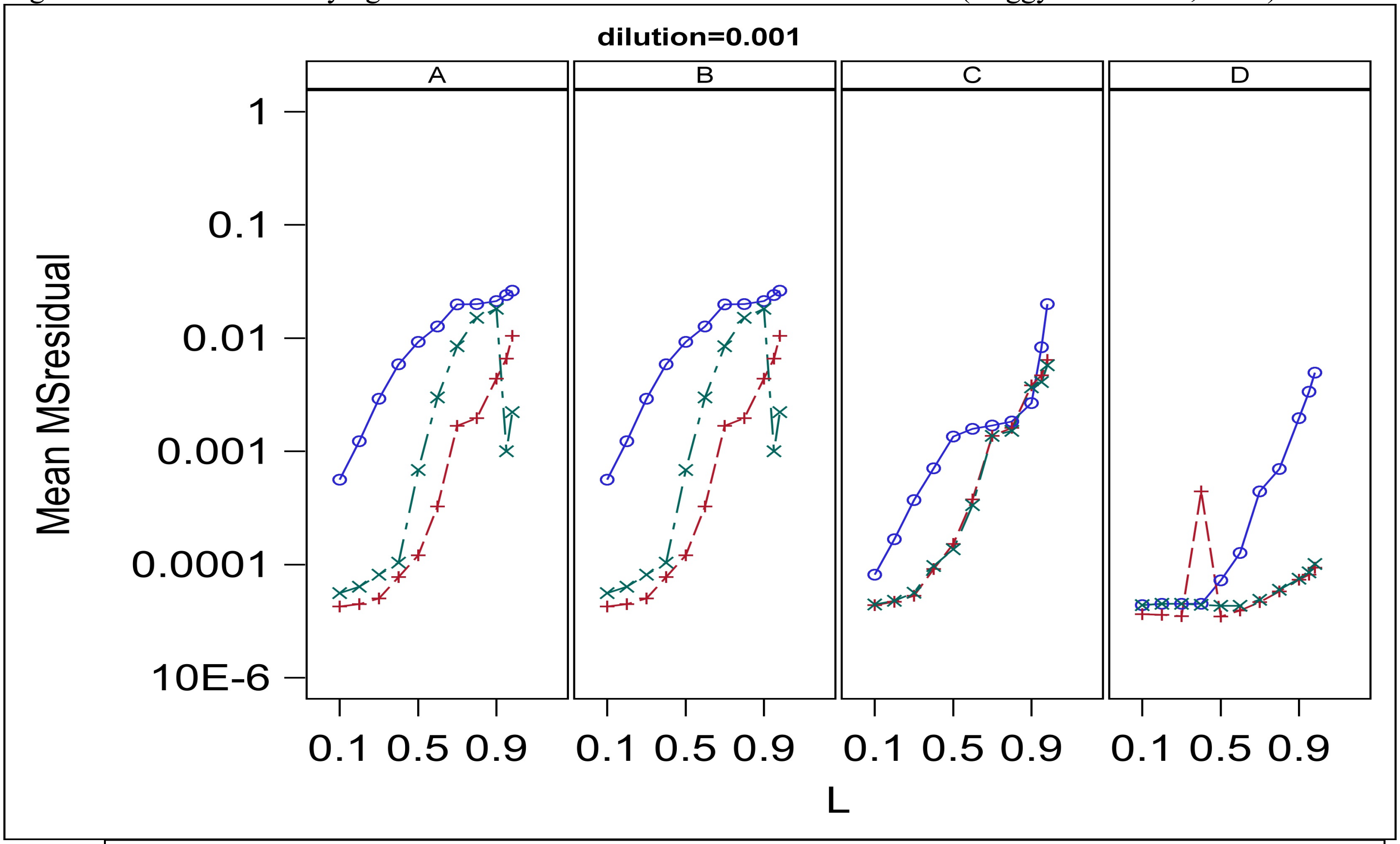

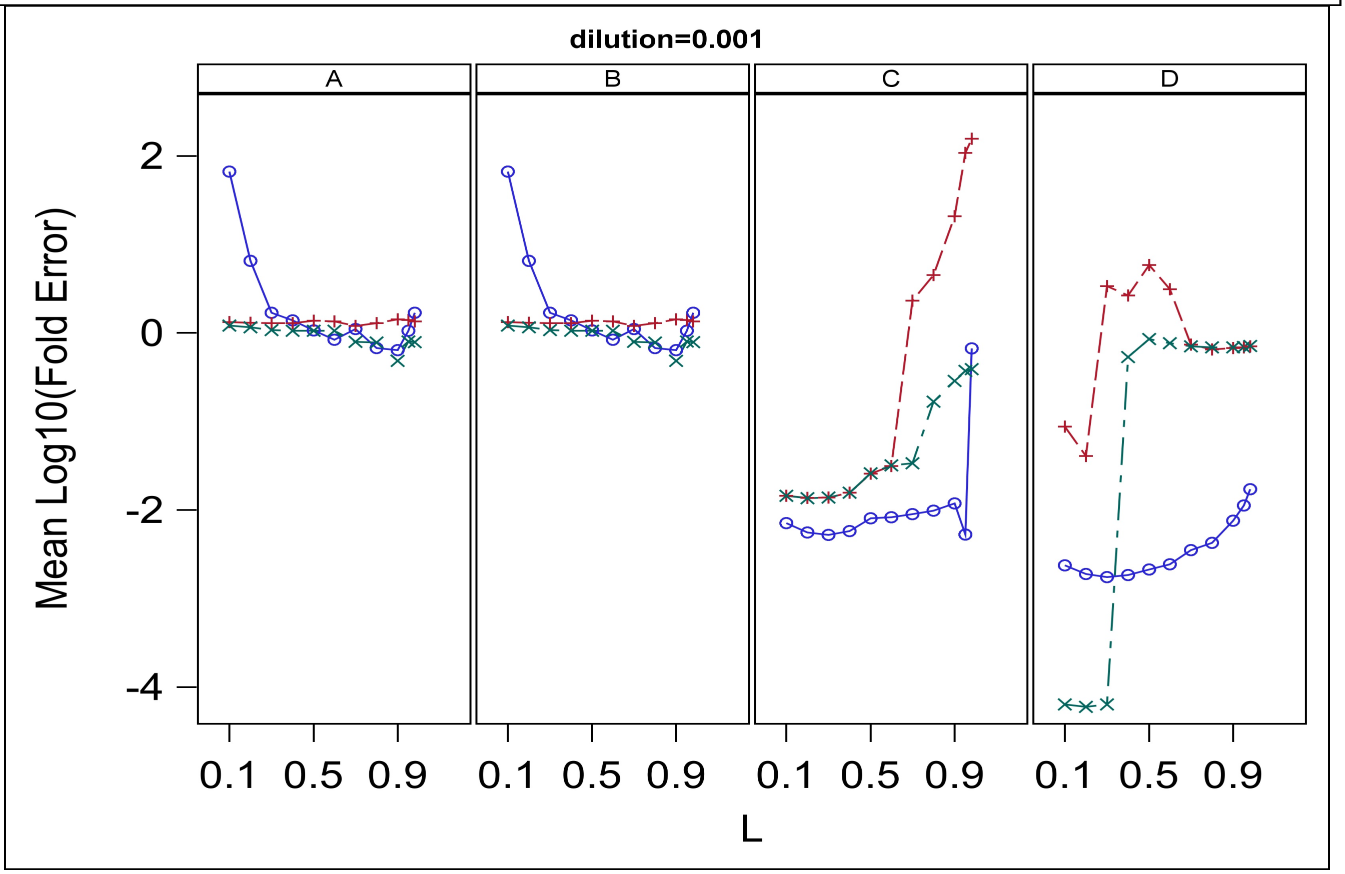

Figure A4-4: Effect of varying L on MSresidual and Fold Error for Dataset 1 (Boggy and Woolf, 2010).

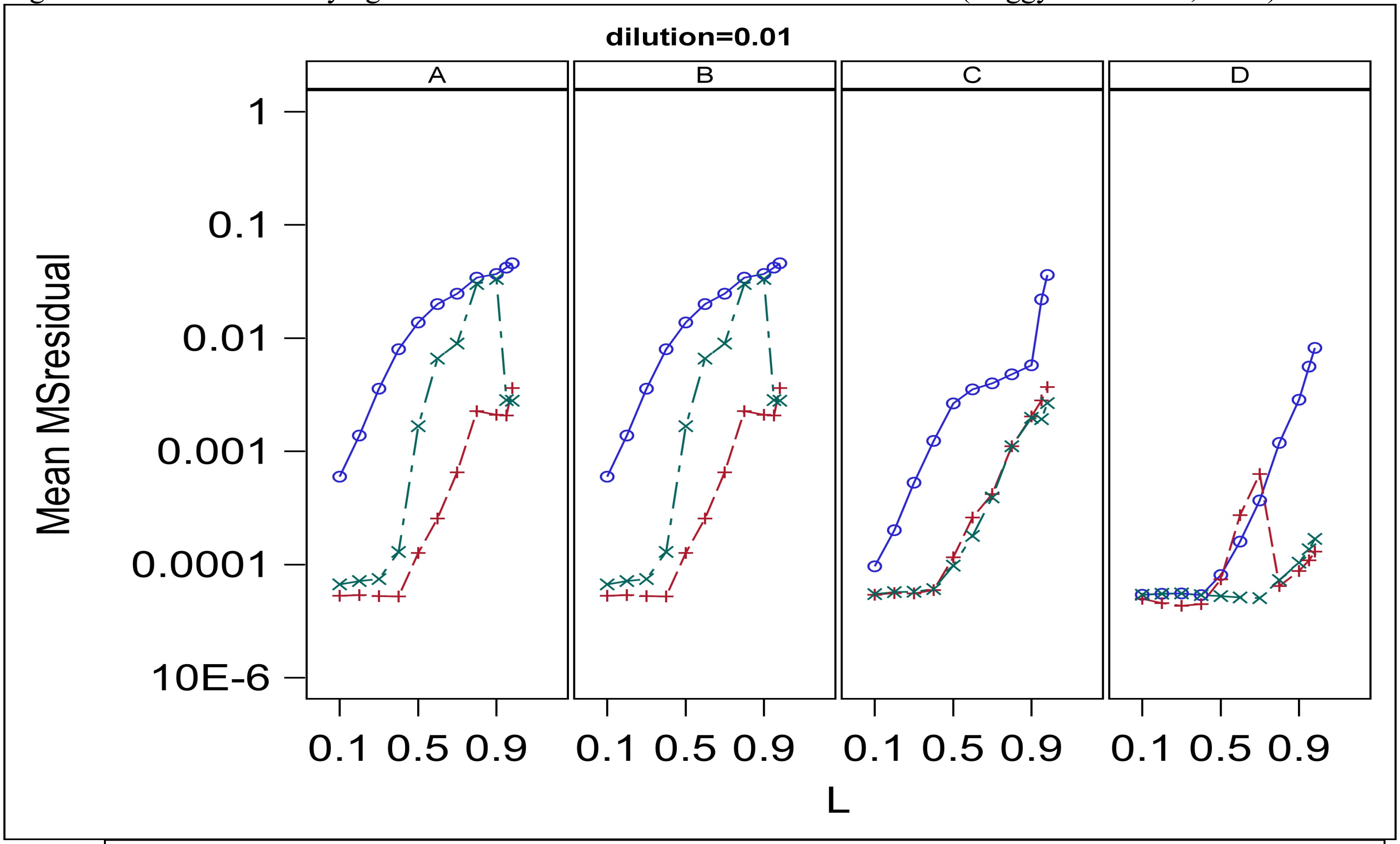

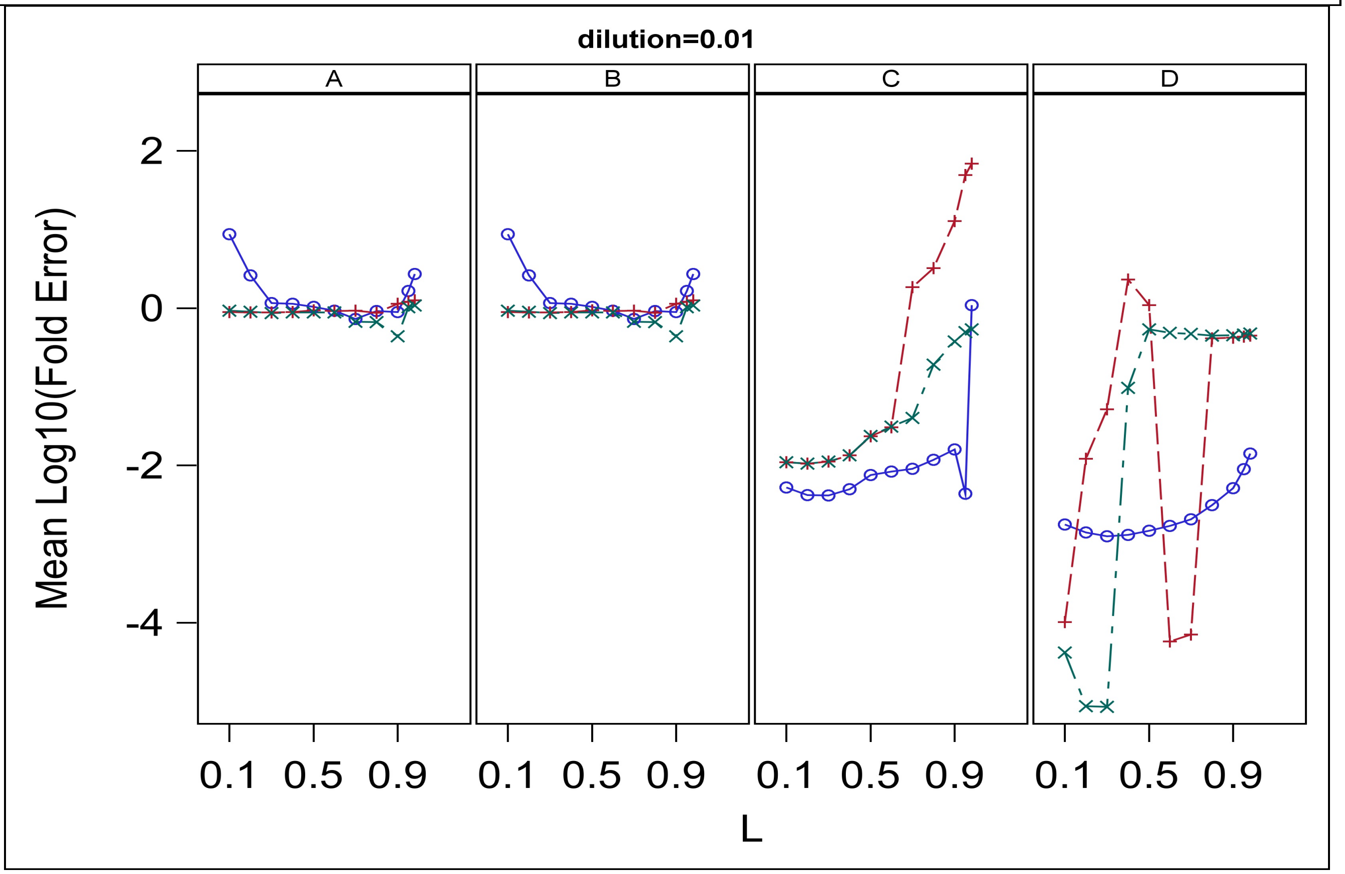

Figure A4-5: Effect of varying L on MSresidual and Fold Error for Dataset 1 (Boggy and Woolf, 2010).

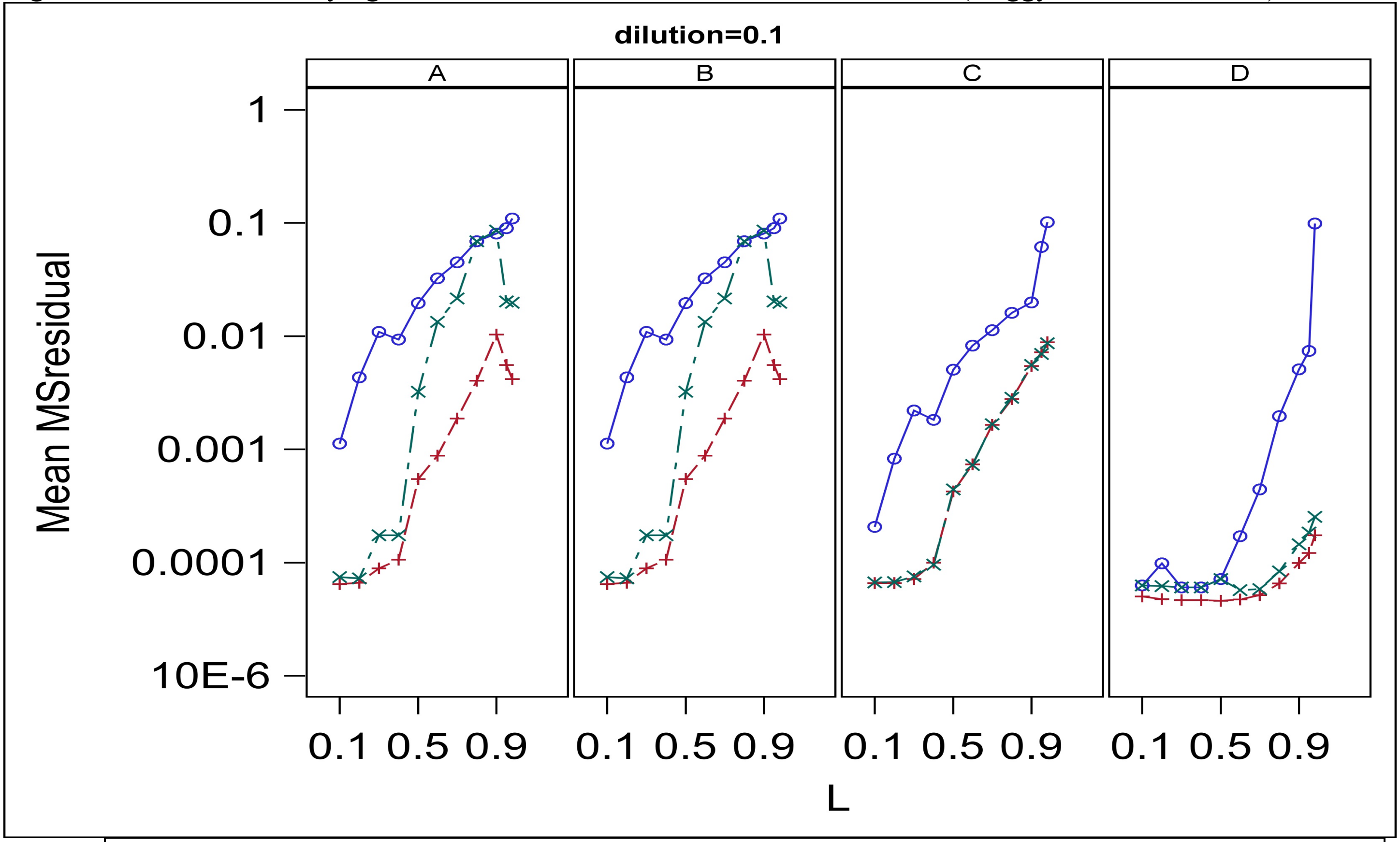

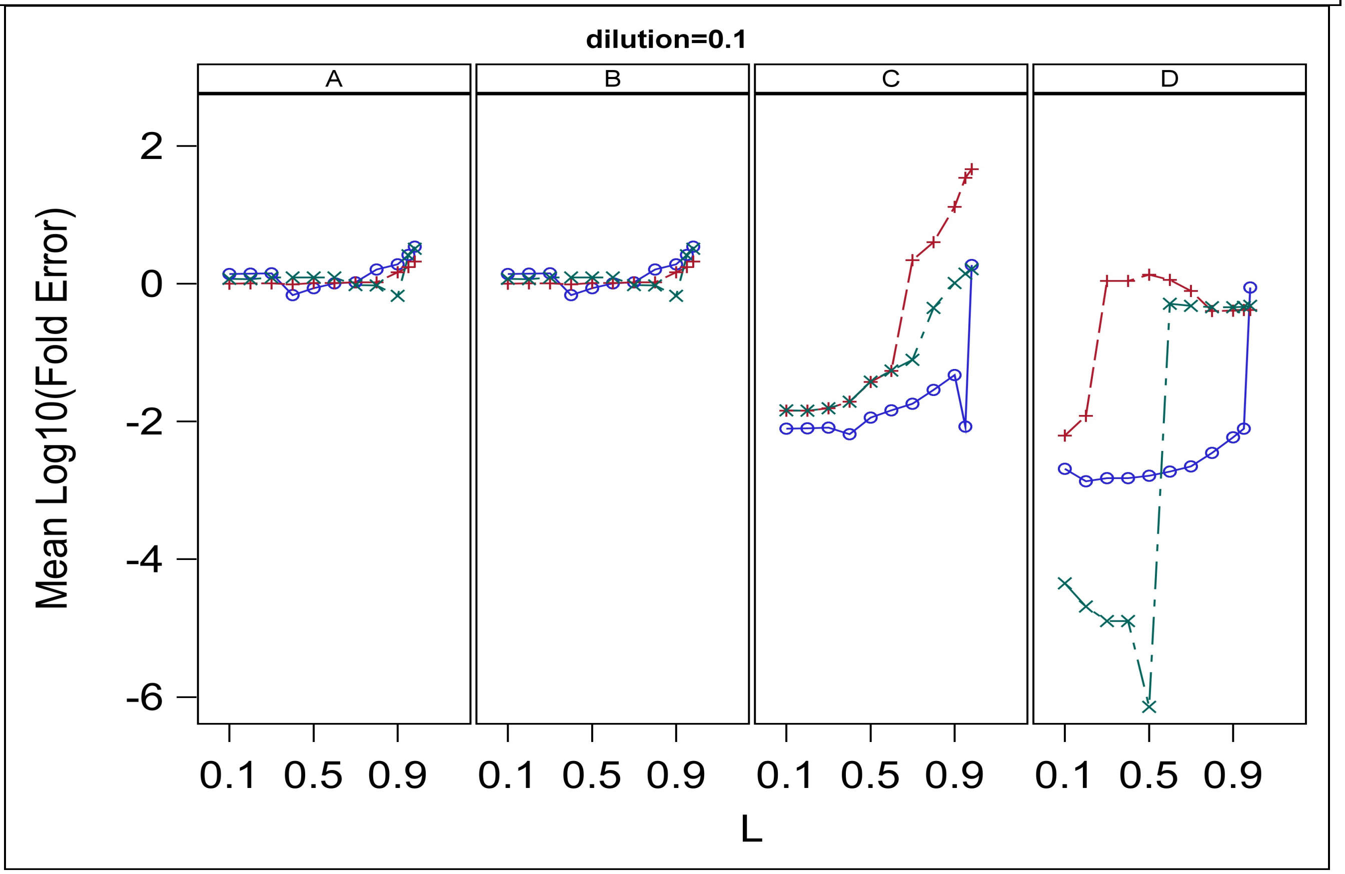

Figure A4-6: Effect of varying L on MSresidual and Fold Error for Dataset 1 (Boggy and Woolf, 2010).

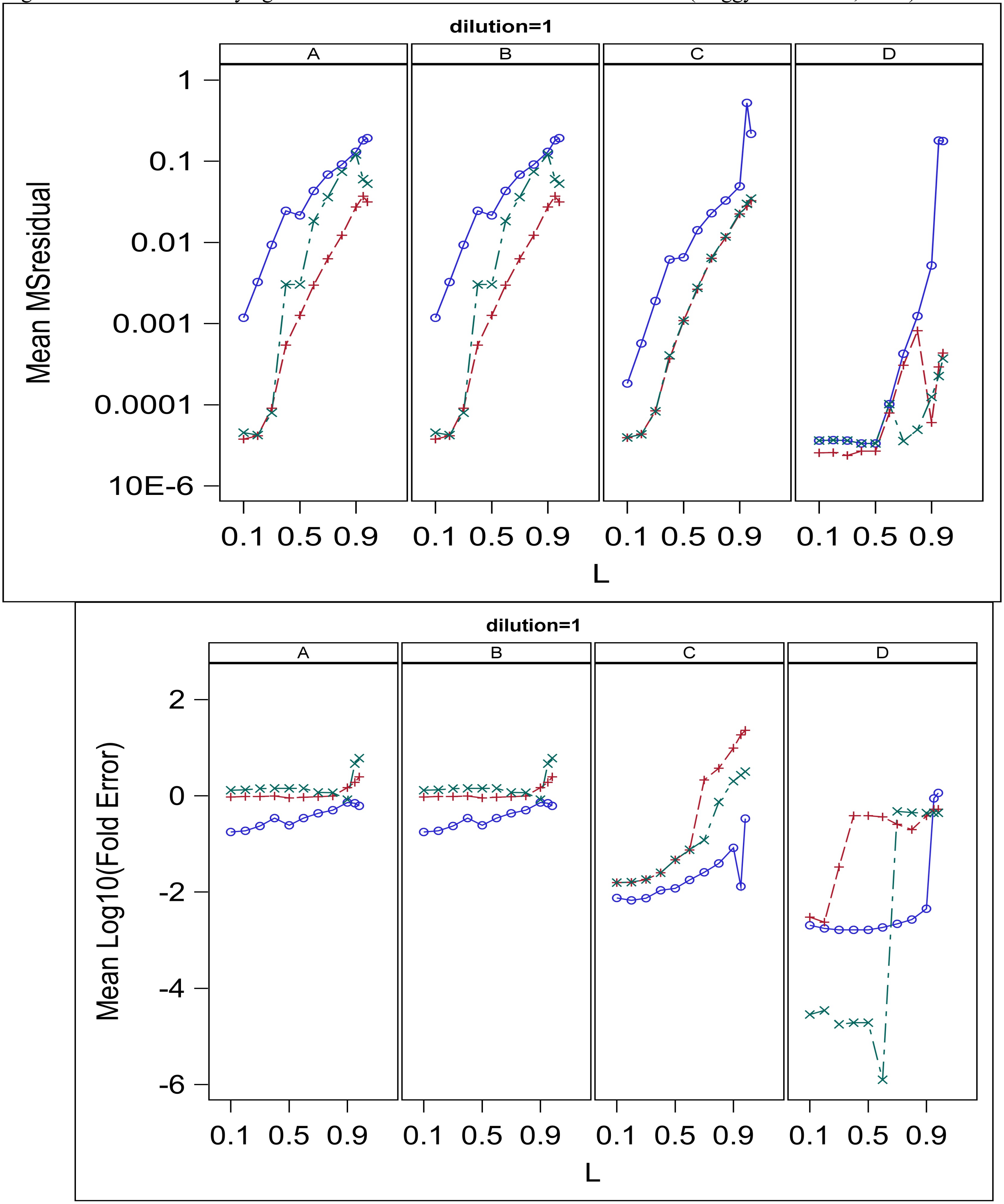

Figure A4-7: Effect of varying L on MSresidual and Fold Error for Dataset 2 (Rutledge, 2004).

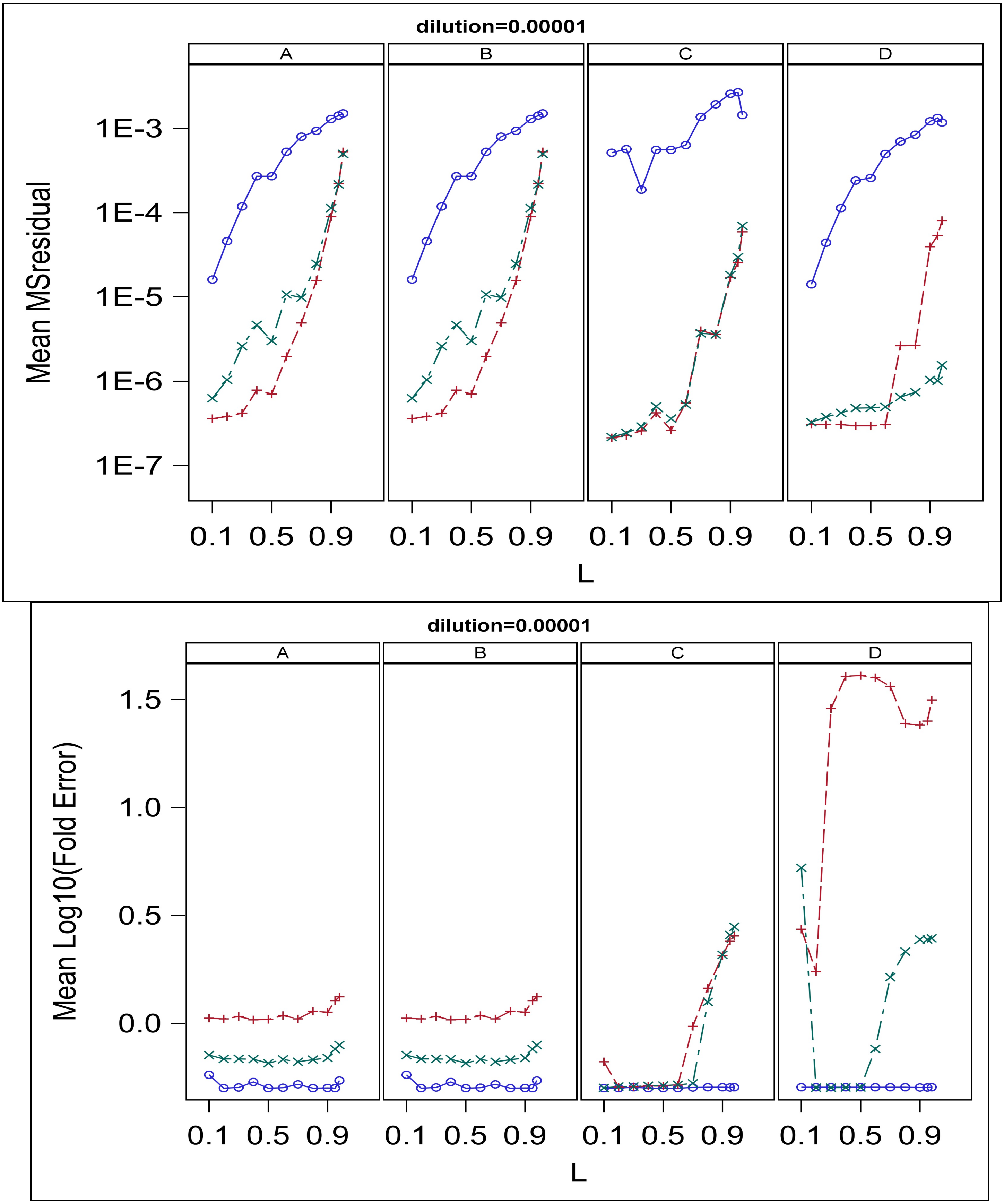

Figure A4-8: Effect of varying L on MSresidual and Fold Error for Dataset 2 (Rutledge, 2004).

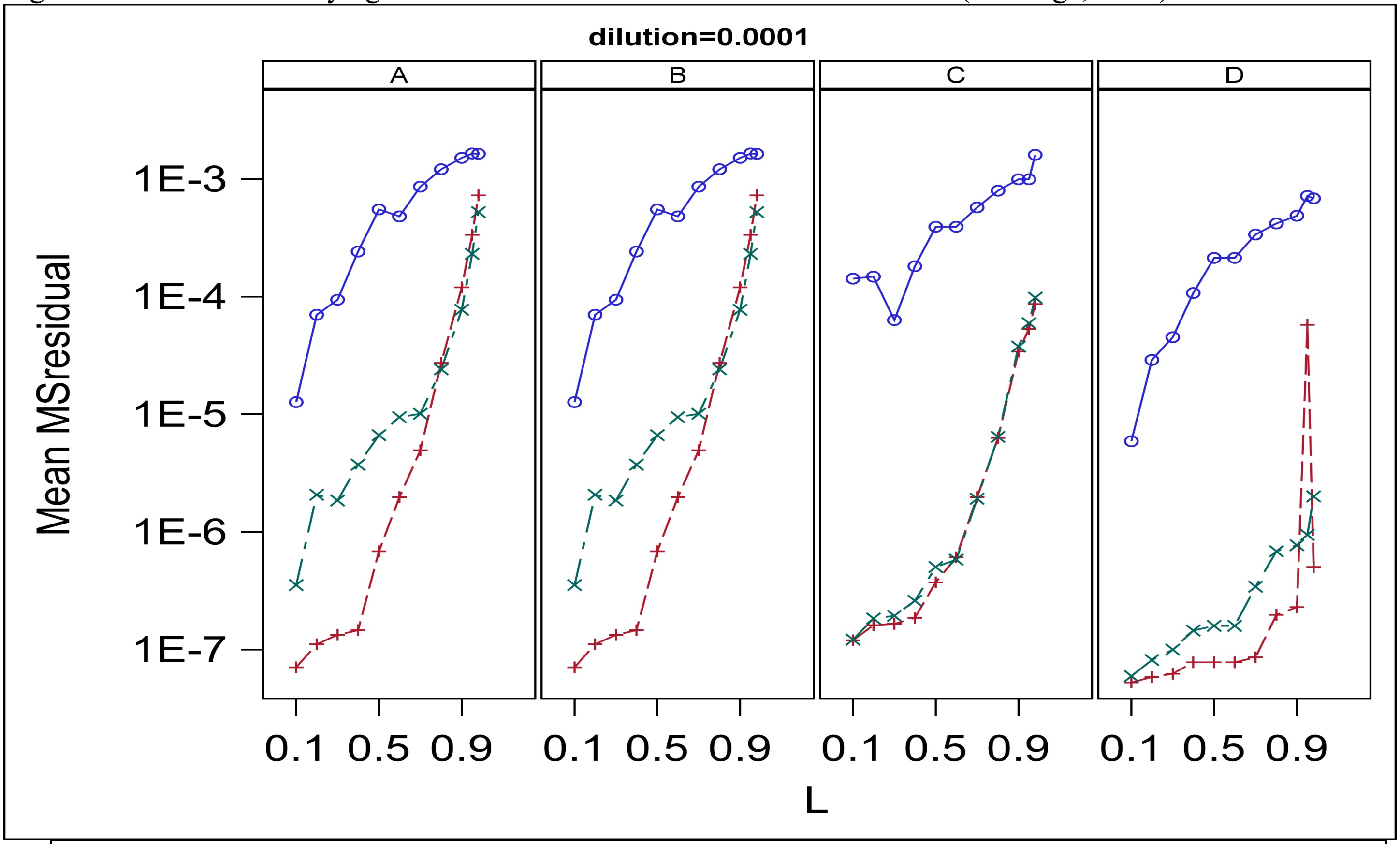

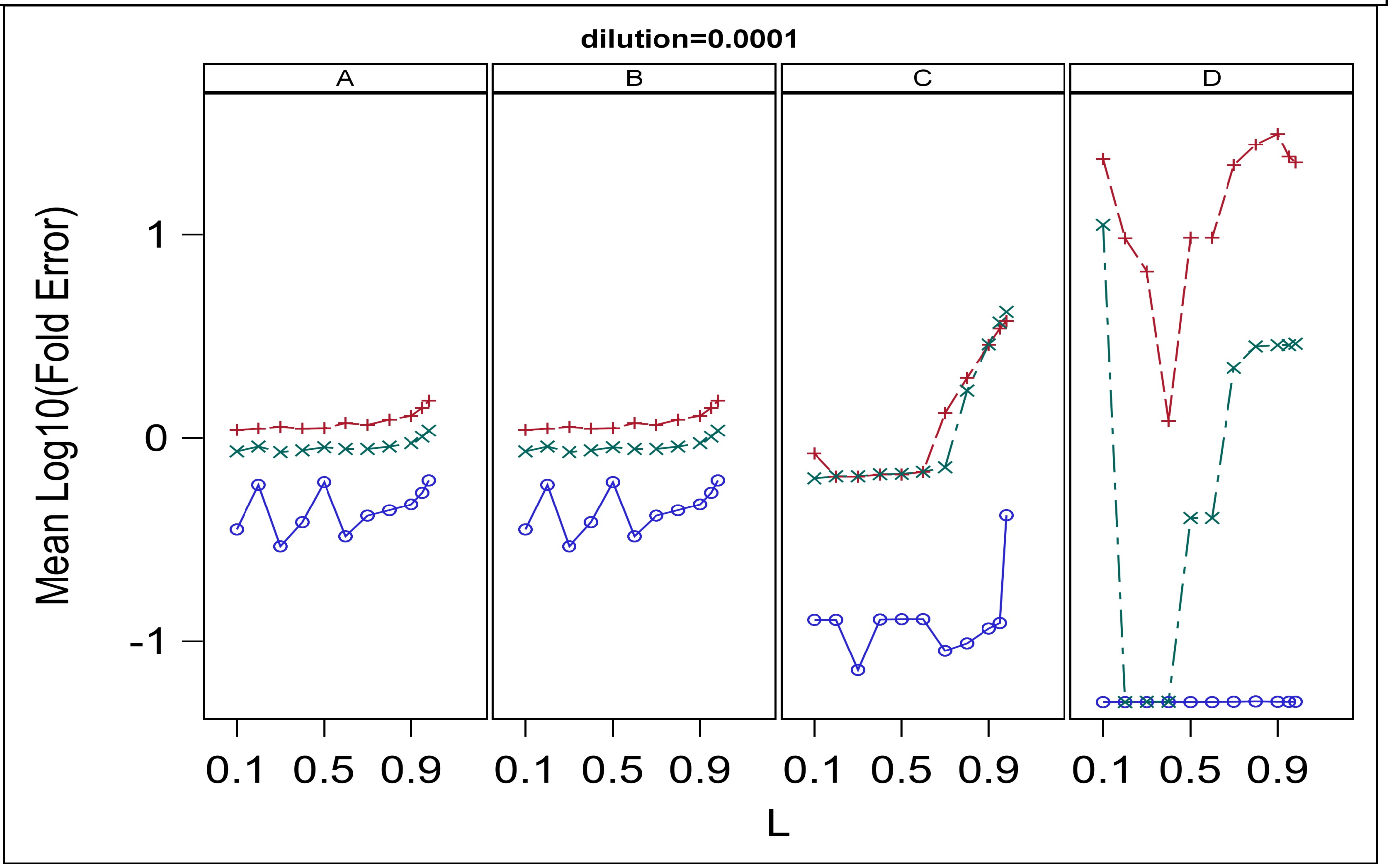

Figure A4-9: Effect of varying L on MSresidual and Fold Error for Dataset 2 (Rutledge, 2004).

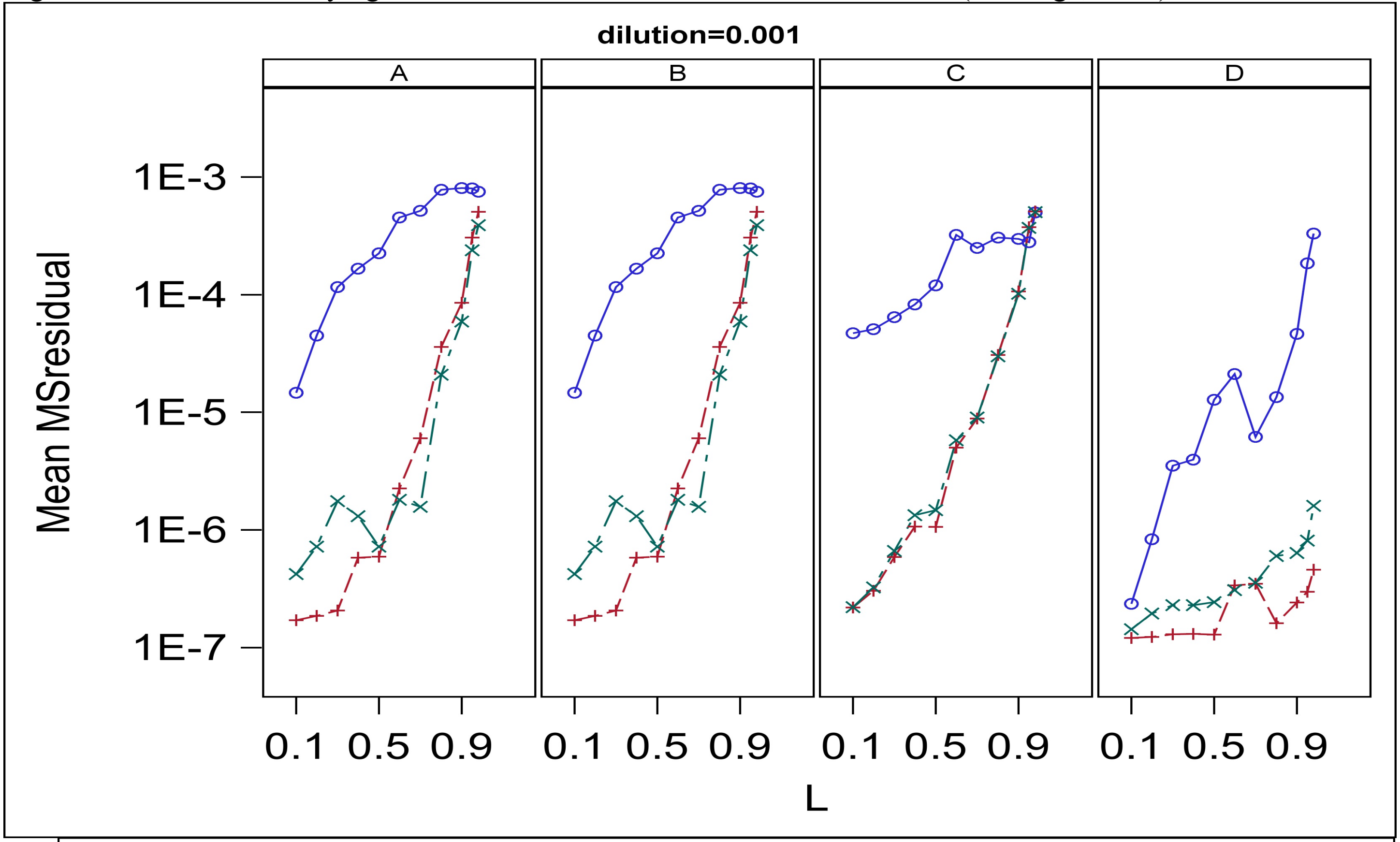

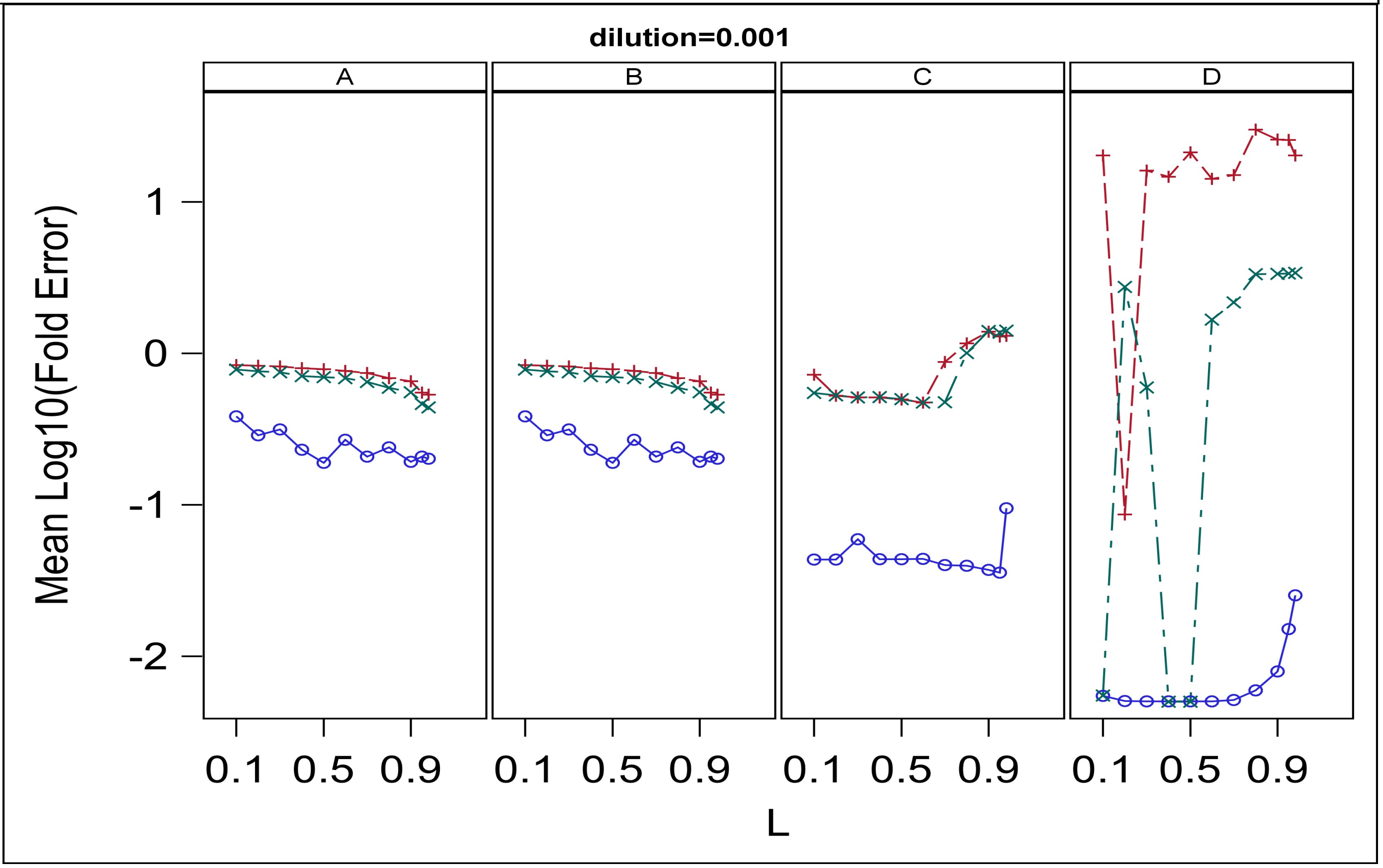

Figure A4-10: Effect of varying L on MSresidual and Fold Error for Dataset 2 (Rutledge, 2004).

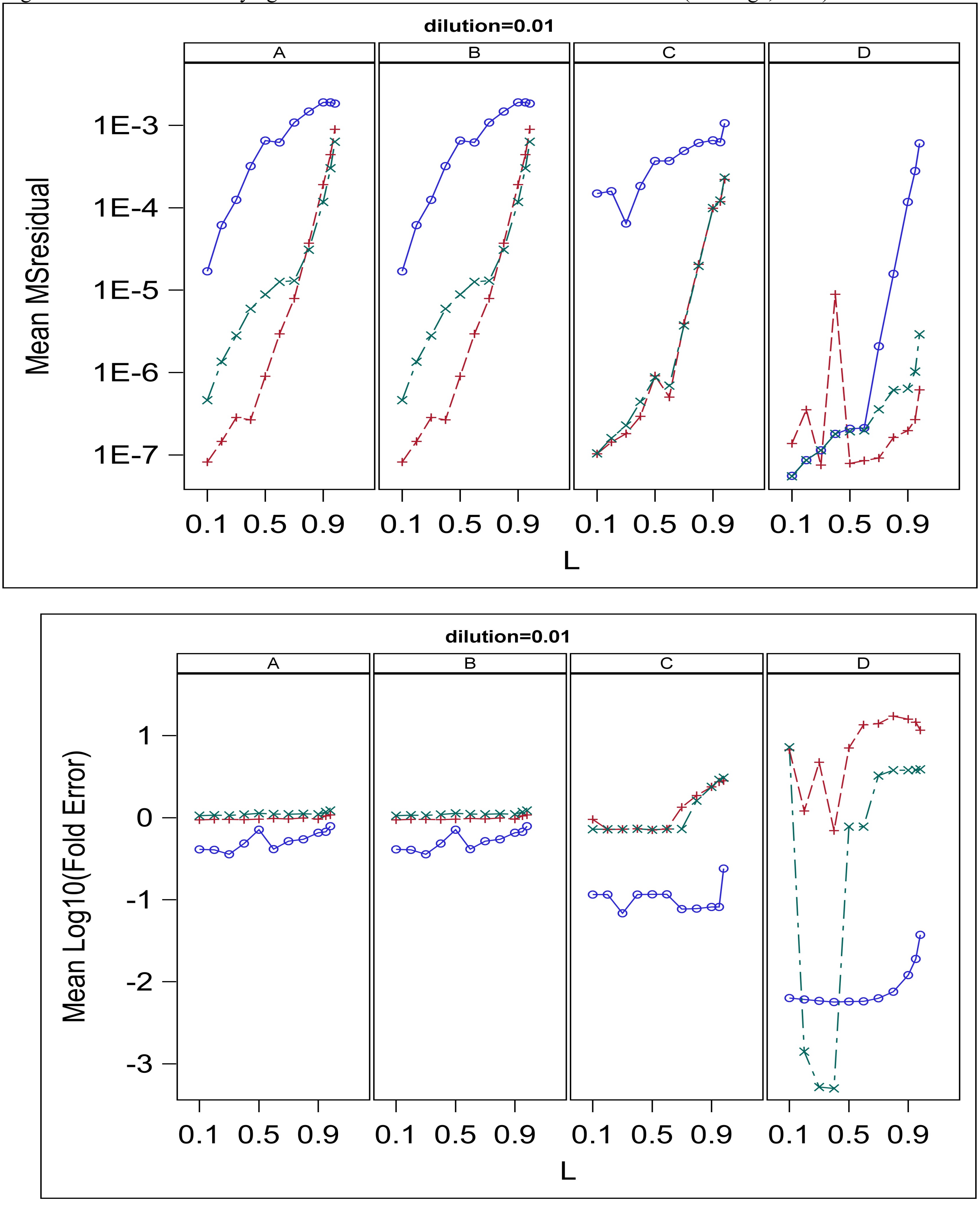

Figure A4-11: Effect of varying L on MSresidual and Fold Error for Dataset 2 (Rutledge, 2004).

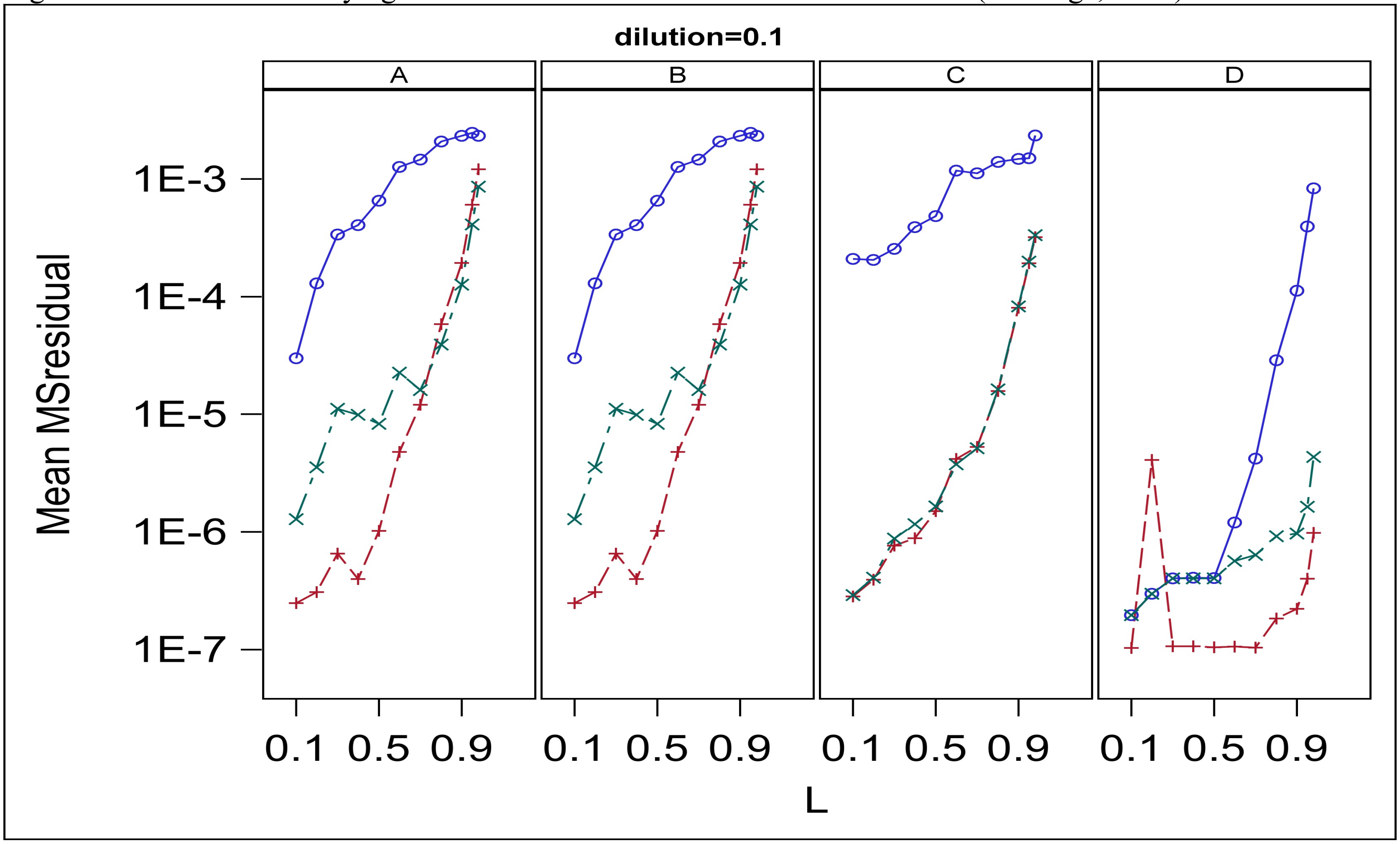

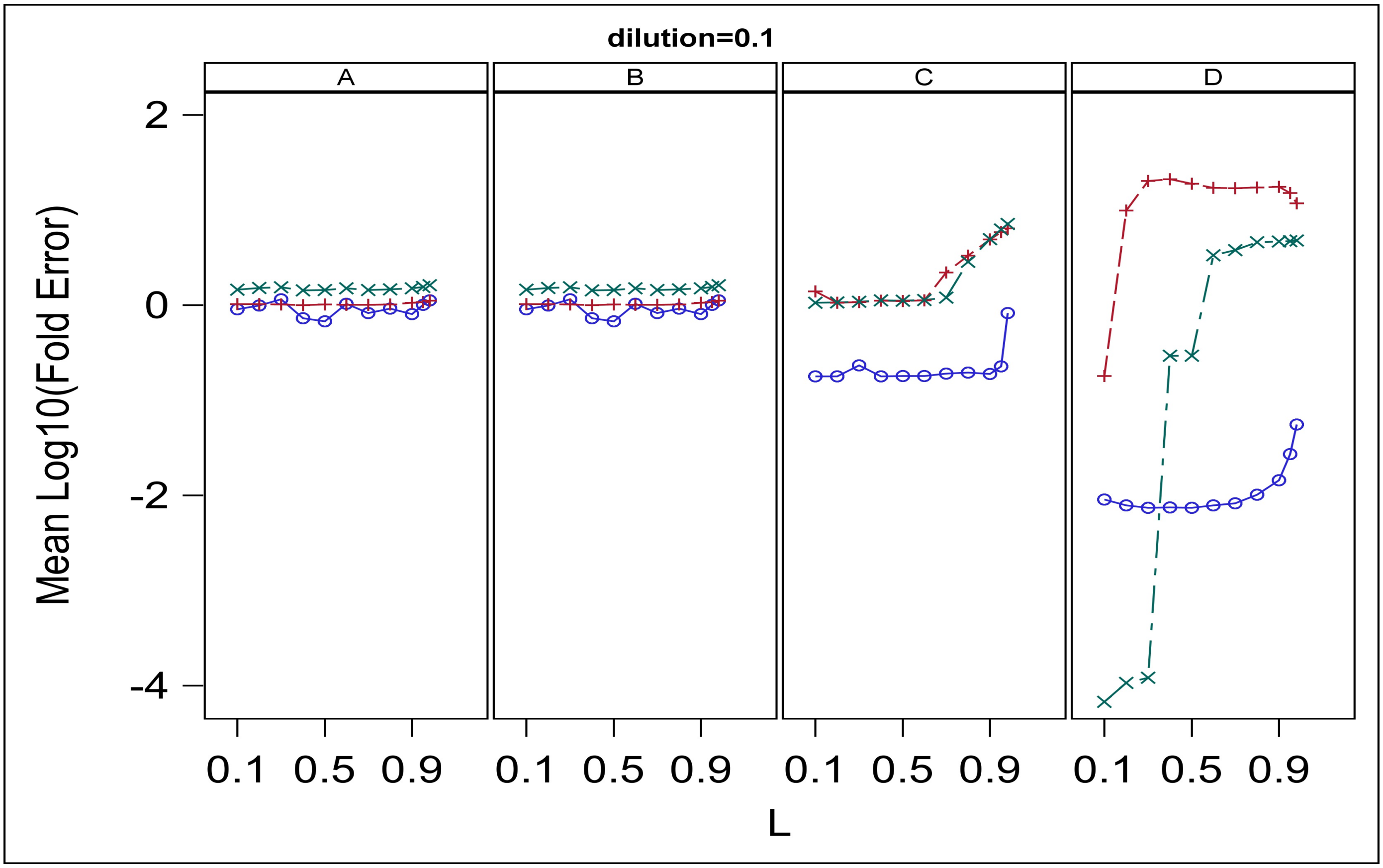

Figure A4-12: Effect of varying L on MSresidual and Fold Error for Dataset 2 (Rutledge, 2004).

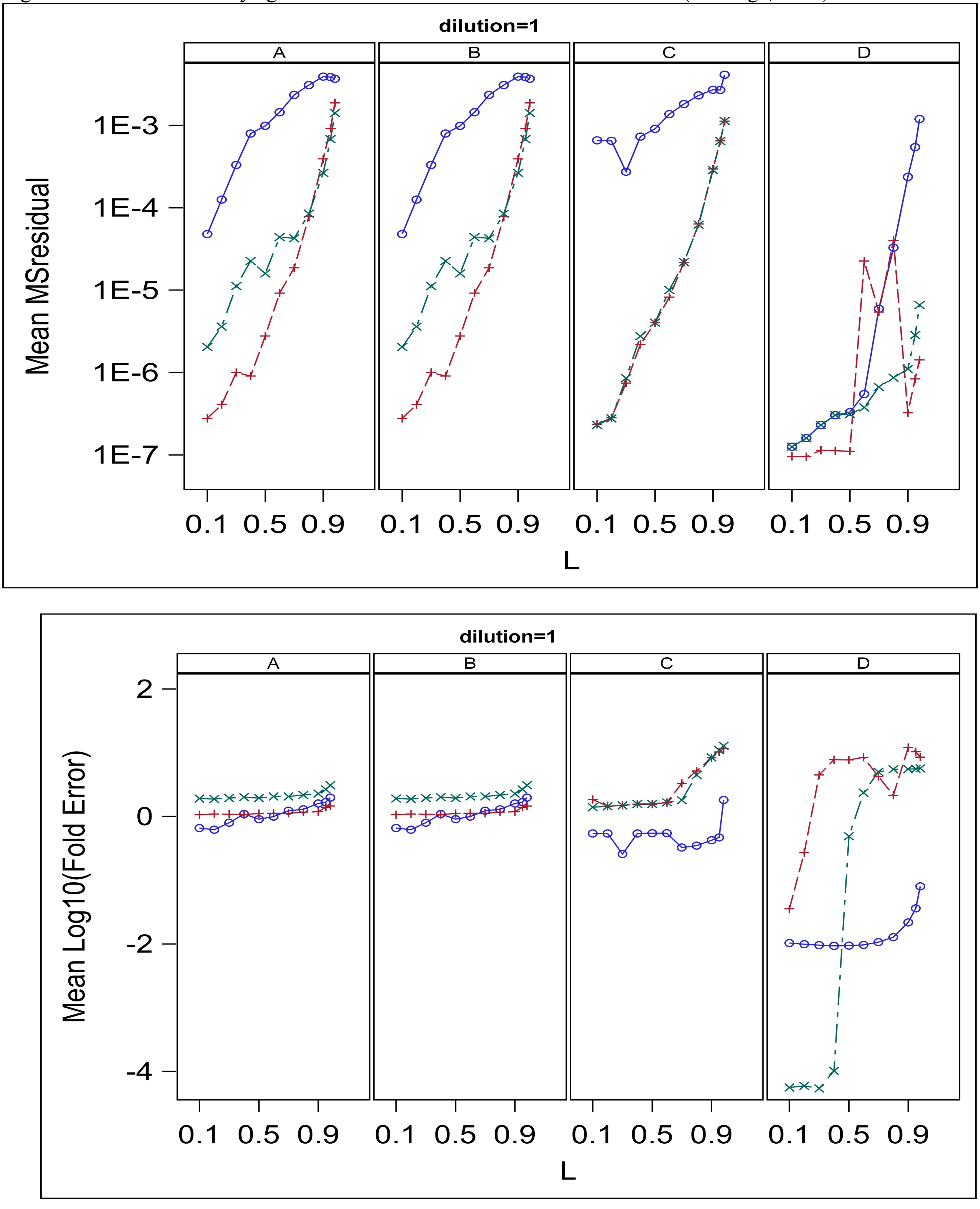

Supplement: Additional file 5 — Effect of varying L on MSresidual and Fold Error. [file 1471-2105-13-203-S5.pdf]
